# Pyrolysis/Non-thermal Plasma/Catalysis Processing of Refuse-Derived Fuel for Upgraded Oil and Gas Production

**DOI:** 10.1007/s12649-024-02866-w

**Published:** 2025-01-08

**Authors:** Maryam Khatibi, Mohamad A. Nahil, Paul T. Williams

**Affiliations:** School of Chemical & Process Engineering, University of Leeds, Leeds, LS2 9JT UK

**Keywords:** Biomass, Plastics, Refuse, Derived fuel (RDF), Non, Thermal plasma, Pyrolysis, Bio, Oil

## Abstract

Refuse-derived fuel (RDF) produced from the processing of municipal solid waste (MSW) has a high content of biomass and plastics. Pyrolysis of RDF produces a bio-oil which is highly oxygenated, viscous, acidic with a high moisture content and unsuitable for direct use in conventional combustion systems and consequently requires upgrading. A novel process of pyrolysis with non-thermal plasma/catalysis has been developed to produce de-oxygenated bio-oils and gases from RDF. The volatiles from the pyrolysis stage are passed directly to a non-thermal plasma/catalytic reactor where upgrading of the pyrolysis volatiles takes place. Detailed analysis of the product oils and gases is presented in relation to process conditions and in the presence of different catalysts (TiO₂, MCM-41, ZSM-5, and Al₂O₃). Even in the absence of a catalyst, the presence of the non-thermal plasma resulted in high yields of CO and CO₂ gases and reduced bio-oil oxygen content, confirming deoxygenation of the RDF pyrolysis volatiles. The addition of catalysts MCM-41 and ZSM-5 generated the highest yields of CO, CO₂, and H₂ due to the synergy between catalyst and plasma. The catalysts ranked in terms of total oxygenated oil yield are as follows: MCM-41 < ZSM-5 < TiO₂ < Al₂O₃. Pyrolysis of RDF produces an oil containing oxygenated species from biomass and hydrocarbon species from plastics. The non-thermal plasma generates high energy electrons which generate radicals and intermediates from the pyrolysis volatiles which synergistically interact with the catalysts to enable deoxygenation of the oxygenated hydrocarbons through decarboxylation and decarbonylation reactions.

**Graphical Abstract:**

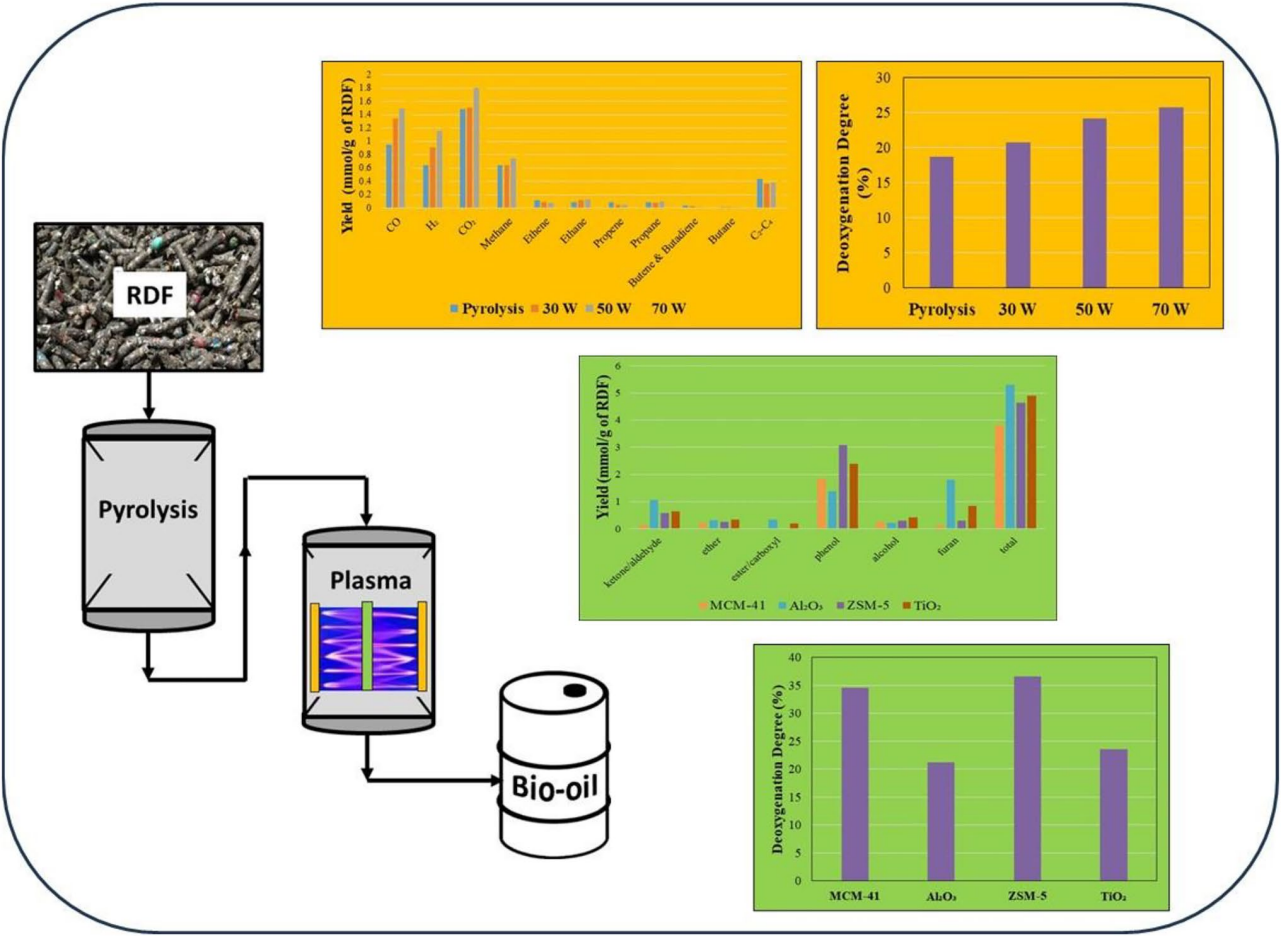

## Statement of Novelty

A novel two-stage reactor system has been used to investigate the pyrolysis with non-thermal plasma/catalytic processing of municipal solid waste in the form of refuse-derived fuel (RDF) with the aim to produce a deoxygenated, upgraded bio-oil. The interaction of the pyrolysis volatiles produced from the biomass and plastic components of the RDF induce synergistic reactions which are enhanced in the plasma/catalytic reaction environment. Detailed gas and bio-oil compositions are presented in relation to the non-thermal plasma processing conditions and the presence of different catalysts types. This work represents for the first time that pyrolysis with non-thermal plasma/catalysis has been presented along with such detailed analysis of the bio-oil and gas products.

## Introduction

Rising energy demand can be met by renewable and sustainable biomass sources due to the lack of supply from non-renewable fossil fuels [1]. Renewable and sustainable fuels, or fuels that can be produced from renewable feedstocks which produce less greenhouse gas emissions, could be the most desirable alternatives [2]. In addition, solid waste management is a global issue that affects everyone [3]. An estimated 2 billion tons of municipal solid waste is generated globally, with over a third of that being uncollected by municipalities. Around 0.74 kg of waste is generated per capita per day [4]. According to the World Bank, municipal solid waste generation is expected to reach 3.4 billion tonnes by 2050 [4]. Refuse-derived fuel (RDF) is a highly combustible fraction of municipal solid waste, which is generated through mechanical biological treatment (MBT). RDF composition is dependent on the source of the MSW and the RDF preparation process, which removes the non-combustible fractions, metals, glass, food waste and fines from the MSW. The resultant RDF is composed of mainly biomass (paper, card, wood) and waste plastics (mainly polypropylene, polyethylene, polyethylene terephthalate and polystyrene) [5].

RDF has a high content of biomass, ranging from 11 to 82 wt.% with a plastic content ranging from 13 to 45 wt% [6]. Pyrolysis of biomass and RDF has been investigated by many authors for the production of bio-oil [7–10]. Bio-oil is a highly viscous black organic liquid with a pH range from 3.5 to 4.2, a smokey odour, and an equivalent energy value of approximately 70–95% to that of petro-crude [10]. Because of its advantages of having a higher energy density and ease of transport and storage, bio-oil is a highly desired liquid fuel compared to solid (bio char) and gaseous (syngas) fuels [1]. However, due to the high content of oxygenated compounds in the oil, its low pH, low heating value, high viscosity, and thermal instability, direct usage of pyrolysis oil derived from mixed feedstock such as RDF is not practicable [2, 5, 11]. As a result, oxygen removal is a critical stage in biofuel upgrading, which can be performed through catalytic pyrolysis and/or a subsequent upgrading step [12].

The application of non-thermal plasma (NTP) processing of pyrolysis gases with a view to upgrading the product bio-oil has been investigated as a novel route to deoxygenate the bio-oil and improve fuel properties [13]. The NTP reaction environment is complex and contains a large number of species capable of inducing chemical reactions such as electron impact ionisation and radical recombination. The average temperature of electrons in non-thermal plasma is much higher than that of gas molecules [14]. Dissociation, electron collision excitation, and ionisation inside the non-thermal plasma yield a range of species, including excited molecules, atomic or molecular ions, metastable species, and neutral atoms. Recombination of the generated reactive chemicals produces neutral molecules with a potentially upgraded product value [15]. The kinetic energy of the charged particles are extremely high. Significant amounts of energy in the form of UV radiation are generated when the ionised species in the plasma recombine with the stripped electrons. The kinetic energy of particles is converted to heat, which can be used to decompose compounds [16]. Non-thermal plasma can be obtained using methods such as dielectric barrier discharge (DBD), corona, gliding arc, and microwave technologies. The DBD system is thought to be more appropriate for most reactions because of its simple design and operation, as well as its good performance under normal operating conditions [14]. The plasma reactor system has several benefits: operation at ambient temperature and atmospheric pressure and operation in a shorter reaction time [14, 17]. A further advantage of the non-thermal plasma system is that in-situ generated hydrogen may be produced from the processing of biomass derived pyrolysis gases. Hydrogen radicals are generated by cracking of the methyl group within the structure of the biomass feedstocks. Since deoxygenation of bio-oils is an essential step in the process of bio-oil upgrading, the in-situ hydrogen production may be used to deoxygenate the bio-oils by hydrogenation, thereby eliminating the need for an external H₂ supply [18].

A further development of the non-thermal plasma reaction environment is the introduction of a catalyst to the process. Non-thermal plasma assisted catalysis combines the effect of the free radicals generated by NTP with catalytic reactions induced by the catalyst [19, 20]. NTP assisted catalysis has the benefits of increasing the catalytic reaction rate, lowering catalyst activation temperature, and minimising catalyst coke deposits [14, 19, 21]. The catalyst comes into direct contact with free radicals, excited atoms, ions, and molecules, which help to accelerate the initiation and propagation of collisions and chemical reactions [22]. The presence of a catalyst has been shown to influence plasma discharge by increasing the electric field, changing the discharge type, and facilitating micro-discharge formation in catalyst pores ^21^. By minimising poisoning and coking, the combination of NTP and catalysts may enhance product selectivity and catalyst stability [15]. The formation of plasma on the surface and within the pores of the catalyst is one of the distinctive aspects of plasma-catalyst interaction, which enhances reaction between reactant species and catalyst [23].

In this work, upgrading of the volatile pyrolysis products from the pyrolysis of refuse-derived fuel (RDF) using a dielectric barrier discharge non-thermal-plasma catalyst reactor was carried out. The aim of the work was to produce a more deoxygenated, higher quality bio-oil. The effect of different input powers in the non-catalytic, non-thermal-plasma upgrading process was initially investigated. In addition, the impact of using different catalysts and the synergistic effect between plasma and catalyst were also evaluated in the pyrolysis/non-thermal plasma/catalytic processes at the optimum input power. To our knowledge, there are no studies focusing on the detailed analysis of bio-oil and gas products for upgrading of the pyrolysis volatiles from the pyrolysis of refuse-derived fuel (RDF) via the interaction between DBD non-thermal plasma and catalyst.

## Materials and Methods

### Materials

Refuse-derived fuel (RDF) was used as the feedstock for the pyrolysis/non-thermal plasma experiments and was supplied from a waste recycling company in the UK (Byker, UK). The RDF was received as compressed pellets of approximately 20 mm diameter × 50 mm long, but were processed into particles measuring 2 mm × 2 mm by shredding and sieving for experimental purposes. Table 1 shows the proximate and ultimate analyses of the refuse-derived fuel (RDF) determined using a Schimadzu TGA-50 thermogravimetric analyser (TGA) and Thermos EA-2000 elemental analyser respectively.

**Table 1:** Proximate and ultimate analyses of refuse-derived fuel (RDF)

Proximate analysis (wt%)	
Volatile	73.662
Fixed carbon	8.165
Ash	14.81
Moisture	3.363
Elemental analysis (wt. %) dry basis	
Carbon	44.09
Hydrogen	5.87
Nitrogen	0.78
Oxygen	33.67
Sulfur	0.26
HHV(MJ/kg)	18.51

The higher heating value (HHV) of the feedstocks was determined by employing Eq. 1 [24].

1 $$\begin{gathered} HHV = 0.3491C + 1.1783H + 0.1005S - 0.1034O - 0.0151N - 0.0211A \left( {\frac{MJ}{{kg}}} \right) \hfill \\ 0\% \le C \le 92.25\% , 0.43\% \le H \le 25.15\% , 0.00\% \le O \le 50.00\% , 0.00\% \le N \le 5.60\% , 0.00\% \le S \le 94.08\% , 0.00\% \le A \le 71.4\% , 4.745 \frac{MJ}{{kg}} \le HHV \le 55.345 \frac{MJ}{{kg}} \hfill \\ \end{gathered}$$

In this context, the symbols C, H, O, N, S, and A represent the carbon, hydrogen, oxygen, nitrogen, sulfur, and ash contents of the materials, respectively. These values are provided as mass percentages on a dry basis [24].

The catalysts utilised for the pyrolysis/non-thermal plasma-catalysis of RDF were MCM-41, Al₂O₃, ZSM-5, and TiO₂. They were crushed and sieved to a particle size of ~ 1 mm. BET nitrogen adsorption was carried out to obtain the surface area of the fresh catalysts using the Brunauer-Emmet-Teller (BET) method. Approximately 0.2 g of each sample underwent degassing at 300 °C under a nitrogen atmosphere for 4 h with a step size of 150 °C starting from 80 °C. The BET analysis of the catalysts gave surface areas of 158, 171, 233, and 734 m^2^ g^−1^ for Al₂O₃, TiO₂, ZSM-5, and MCM-41, respectively.

## Experimental Reactor System

**Fig. 1:**
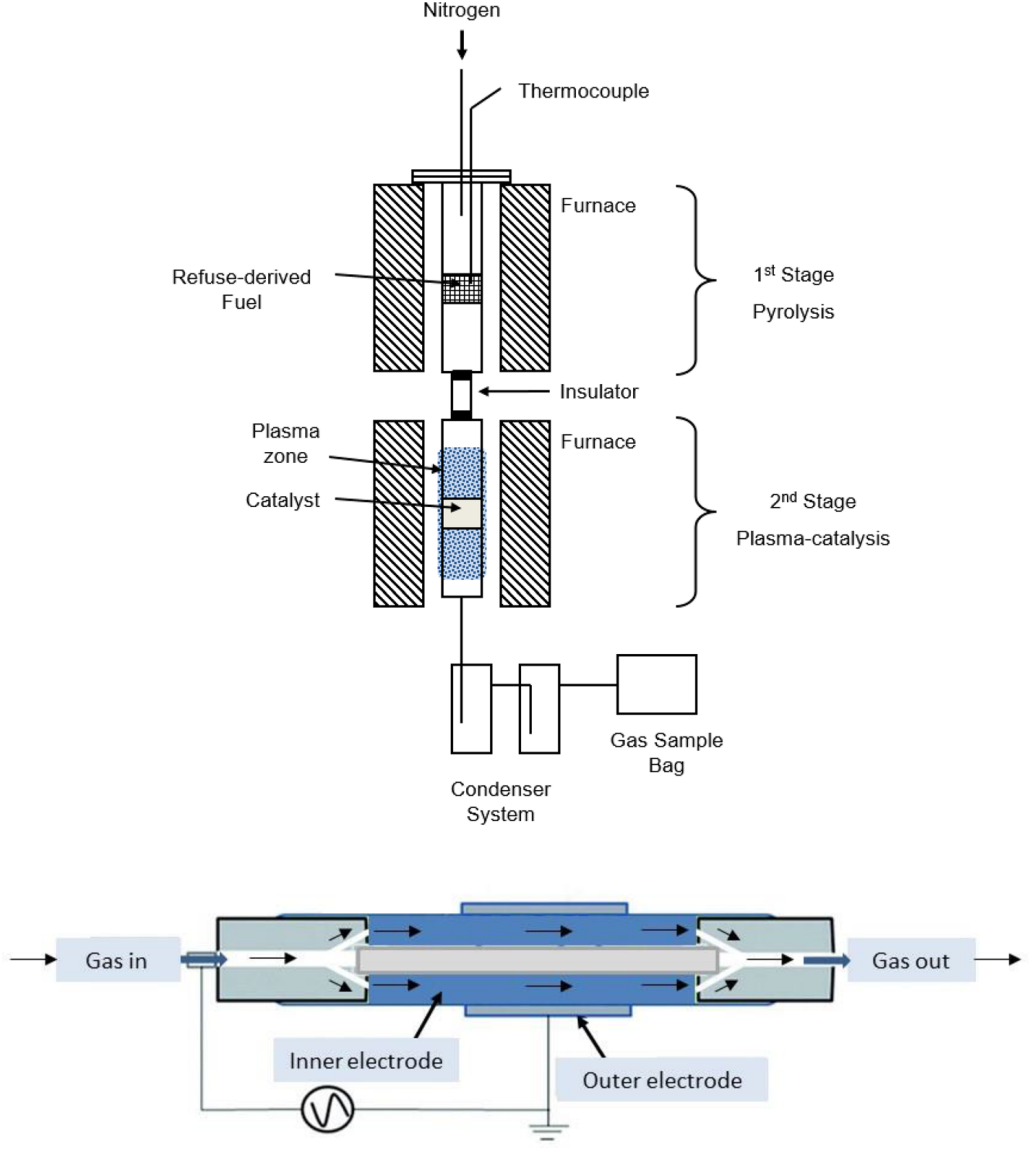
**a** Schematic diagram of the pyrolysis/non-thermal plasma catalyst reactor system **b** Schematic diagram of non-thermal plasma-catalysis reactor

The pyrolysis/non-thermal plasma experiments were conducted using a two-stage reaction system, as depicted in Fig. 1a. In the initial stage, the refuse-derived fuel underwent pyrolysis reactions. The resulting pyrolysis volatiles were then passed directly to the second stage DBD non-thermal plasma reactor for plasma upgrading. Throughout the process, the reactor system was consistently purged with nitrogen gas. The pyrolysis reactor was made of stainless steel with dimensions of 250 mm in length and 20 mm in internal diameter. It was encased and heated by an electric tubular heating furnace with temperature control and monitoring, ensuring precise temperature control and even heat distribution. Within the pyrolysis reactor, the feedstock was contained in a stainless-steel crucible suspended from the reactor lid. An electric insulator tube separated the pyrolysis reactor from the plasma reactor. The pyrolysis products were transferred to the second stage DBD non-thermal plasma reactor by nitrogen purge gas.

The plasma reactor employed in this study was constructed from quartz glass tube and was a coaxial Dielectric Barrier Discharge (DBD) plasma reactor, featuring a dual-electrode configuration as illustrated in Fig. 1b. The first electrode, serving as the inner electrode was 254 mm long and 18 mm diameter and consisted of a stainless steel rod, connected to the power supply and positioned at the reactor centre. The second electrode, acting as the outer electrode, was a 95 mm long copper mesh, which served as the low voltage electrode and was wrapped around the 23 mm diameter quartz glass tube. This glass tube acted as a dielectric material, separating the inner and outer electrodes. The region where plasma reactions occurred, known as the discharge zone, spanned 95 mm along the axial length and featured a 5 mm discharge gap between the two electrodes. To power the DBD plasma reactor, an AC high-voltage power supply with a frequency of 1500 Hz and a maximum peak-to-peak voltage of 20 kV was employed. The high voltage was applied to the inner electrode, while the outer electrode was grounded. The discharge process was monitored using a digital oscilloscope. In this non-thermal plasma reactor system, the applied electric field ionized nitrogen molecules within the discharge zone, generating electrons that collided with the molecular species derived from pyrolysis within the discharge gap. This collision led to the production of reactive components that could facilitate the initiation and progression of chemical reactions.

The gases produced in the DBD reactor were passed through a series of glass condensers, consisting of an air-cooled condenser and then a dry-ice cooled glass condenser. Non-condensable gases were transferred to a 25 L Tedlar™ gas sample bag for further analysis. Experiments under the same conditions was repeated several times to determine the repeatability and efficiency of the experimental equipment and procedure using RDF as the feedstock under pyrolysis-plasma/catalysis conditions. The repeatability of the experiments could be evaluated using the standard deviation which was gas, 0.031, liquid, 0.047 and char 0.01 and relative standard deviation which was, gas, 6.57%, liquid 6.24% and char 3.02%. The reliability of gas composition and concentration determination was also evaluated, for example, the relative standard deviation for H_2_ gas concentration was 0.63%.

The yields of the products were determined by applying Eq. 2, Eq. 3 and Eq. 4, and the masses of the gas compounds were computed based on the ideal gas law. Equation 5 was used to compute the degree of deoxygenation.

2 $$Gas\,yield (\%)=\frac{mass\,of\,gas}{mass\,of\,feedstock} \times 100$$

3 $$Char\,yield (\%)=\frac{mass\,of\,char}{mass\,of\,feedstock} \times 100$$

4 $$Liquid\,yield \left(\%\right)=100-Gas\,yield \left(\%\right)-Char\,yield (\%)$$

5 $$Deoxygenation\,degree \left(\%\right)=\frac{mass\,of\,oxygen\,in\,gas}{mass\,of\,oxygen\,in\,RDF}\times 100$$

## Gas Analysis

The product gases underwent analysis via packed column gas chromatography (GC) utilizing a series of Varian 3380C gas chromatographs to ascertain their gas composition and concentration. To identify the permanent gas compositions, such as H₂, O_2_, CO, and N_2_, a GC with thermal conductivity detection (GC-TCD) was employed. Argon served as the carrier gas, and a molecular sieve column with a packing with particle size ranging from 60 to 80 mesh was utilized. Furthermore, GC-TCD was employed to assess the presence of CO₂ in the product gas a second Varian GC. Argon served as the carrier gas, and the column was packed with HayeSep material of 60–80 mesh. For the determination of hydrocarbons ranging from C_1_ to C_4_, gas chromatography with flame ionization detection (GC-FID) was utilized in a third GC. Nitrogen was employed as the carrier gas, and an 80–100 mesh HayeSep packed column was used. The chromatographic peaks were quantified using Harley Peakmaster Integration software.

## Oil Analysis

The composition of the product oil was examined using gas chromatography-mass spectrometry (GC/MS), employing a Hewlett Packard 5280 GC combined with an HP 5271 ion trap mass spectrometric detector. In the GC/MS setup, a Restek RTX-5MS column measuring 30 m in length, with an internal diameter of 0.25 mm and a film thickness of 25 μm consisting of fused silica with 95% dimethyl polysiloxane and 5% diphenyl solid phase, was utilized. Helium served as the carrier gas. Before injection into the GC/MS, the oils were dissolved in dichloromethane. The identification and quantification of compounds in the oil were accomplished by analyzing the total ion chromatographic peaks in relation to their retention times. This analysis utilized the NIST 2008 spectral library and a mass spectral similarity index of > 70% was employed for the identification of compounds. Concentration and mass of each compound were calculated based on Eq. 6 and Eq. 7.

6 $$concentration\,of\,compound\,x=\frac{peak\,area\,of\,compound\,x}{peak\,area\,of\,standard }\times concentration\,of\,standard$$

7 $$mass\,of\,compound x=\frac{concentration\,of\,compound\,x}{total\,concentration}\times mass\,of\,produced\,liquid$$

Elemental analysis of oils were carried out using an elemental analyser (CHNS-O) using a Thermo EA2000 instrument to measure the amount of carbon, hydrogen, nitrogen, sulphur, and oxygen. The samples were placed in a 900 °C furnace with a constant flow of helium. The samples were combusted after being exposed to pure oxygen for a few seconds. The combustion products were separated using a gas chromatography column and then detected using thermal conductivity detection after passing through an oxidation/reduction reactor to ensure combustion products are in the correct form (CO₂, H₂O, N_2_, and SO_2_).

## Catalyst Characterisation

Temperature programmed oxidation (TPO) of the used catalysts was carried out to identify the features of the carbonaceous coke deposits produced on the catalyst during the reaction. TPO involves the oxidation of the coke that has accumulated on the catalyst, and this is achieved by exposing it to air while controlling the heating rate. This process was carried out using a TGA-50 Shimadzu instrument measuring the amount of carbon oxidized as a function of temperature. The TGA temperature program was set to heat the catalysts from room temperature to 800 °C at a heating rate of 15 °C min^−1^. Scanning electron microscopy (SEM) was performed on the catalyst samples using a Carl Zeiss EVO MA15 microscope set to 2.0 kV accelerating voltage. The fresh catalysts were characterized by X-ray diffraction (XRD) using XRD Bruker D8 diffractometer using Cu-Kα radiation. The results were recorded over 2θ in the range of 10–80° and a scanning step of 0.033.

## Results and Discussion

### Thermal Decomposition of Waste Components

Initial work was carried out to determine the thermal decomposition characteristics of the refuse-derived fuel (RDF) using thermogravimetric analysis (TGA). In addition, to further understand the thermal degradation interactions of the biomass and plastic components of the RDF, individual biomass (wood, newspaper, and cardboard), individual plastics (polypropylene (PP), polystyrene (PS), polyethylene terephthalate (PET), low-density polyethylene (LDPE) and high-density polyethylene (HDPE)), and different mixtures of biomass and plastic were analysed by TGA to investigate their thermal degradation behaviour in comparison with the RDF sample. The weight percentage of biomass and plastic in the mixtures was 0, 25, 50, 75, and 100%. The biomass in the mixture samples consisted of 16% wt. wood, 42% wt. newspaper, 42% wt. cardboard and the plastic content in the mixture samples was composed of 22% wt. PP, 22% wt. PS, 21.5% wt. HDPE, 21.5% wt. LDPE, 13% wt. PET. This content of plastics was chjosen to represent a typical mixture of plastics found in municipal solid waste and hence in typical RDF. Thermogravimetric analysis was carried out using a Shimadzu TGA-50. The samples were ground to powder using a Cryomill to prepare them for analysis. About 20 mg of each sample was weighed in an alumina pan and the TGA temperature program was set to heat the sample from room temperature to 650 °C at a heating rate of 10 °C min^−1^ and followed by a hold time of 20 min. The heating atmosphere was nitrogen with a flow rate of 50 ml.min.^−1^

**Fig. 2:**
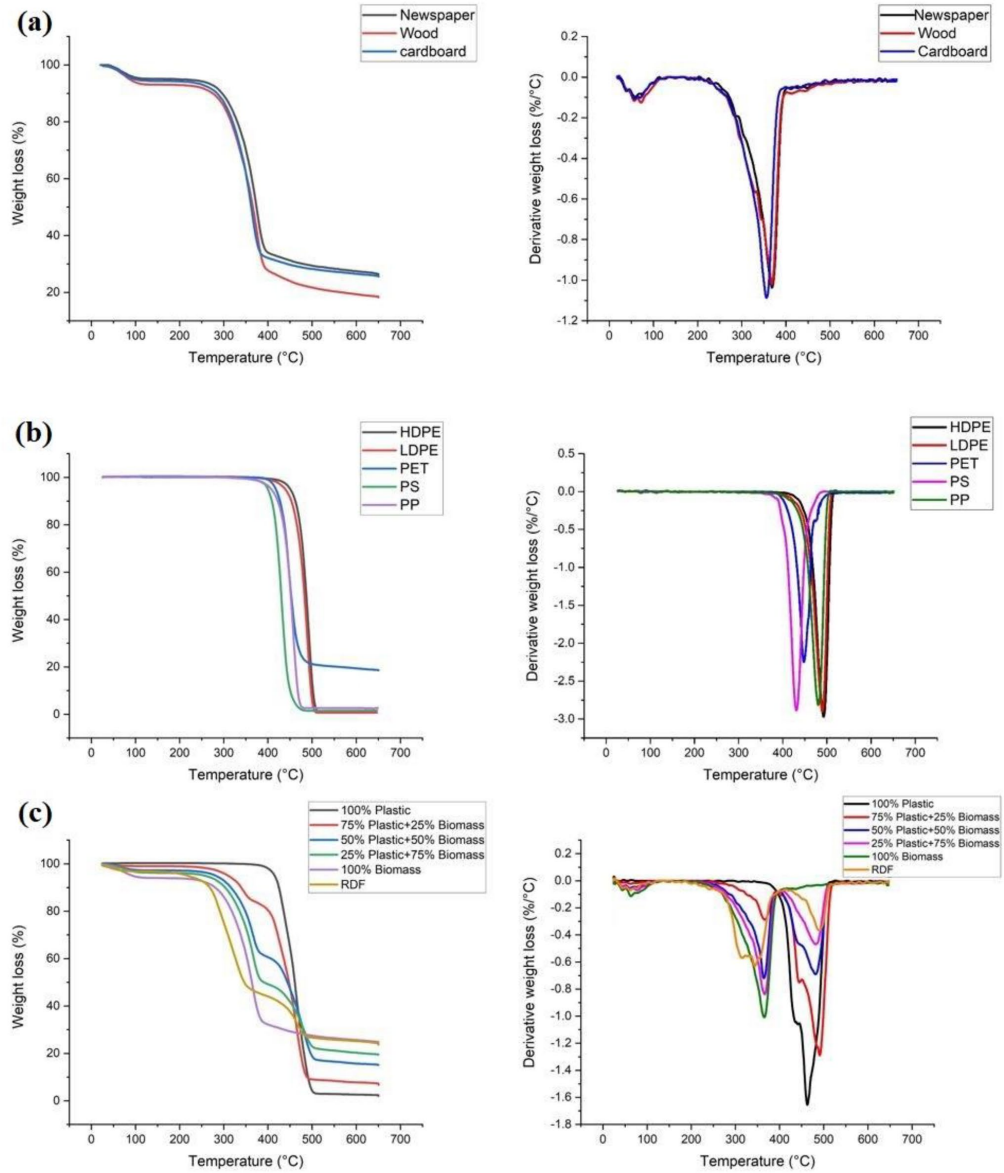
TGA-DTG thermograms of **a** newspaper, wood, cardboard, **b** HDPE, LDPE, PET, PP, and PS, **c** RDF and different biomass/plastic mixtures

Figure 2 shows the thermogravimetric analysis (TGA) thermograms and derivative thermogravimetry (DTG), representing the rate of weight loss of the individual biomass samples, individual plastics, RDF, and mixtures of biomass and plastic.

Biomass: Fig. 2a shows that all the biomass types thermally decomposed in the temperature range of 220 °C–400 °C with a weight loss of 60% for newspaper and cardboard, and 65% for wood. The peak of decomposition occurred at around 370 °C with a weight loss rate of 1.1%/°C at this temperature. Biomass is composed of cellulose, hemicellulose, and lignin and thermal degradation of hemicellulose occurs between temperatures of 250 °C and 350 °C, while cellulose decomposition proceeds between 325 °C and 400 °C. Lignin decomposition, on the other hand, occurs throughout the pyrolysis temperature range [25]. Thermogravimetric analysis reveals that biomass degradation typically occured in three main stages: moisture evolution, hemicellulose-cellulose decomposition, and lignin degradation [26]. El-Sayed et al. conducted TGA to investigate the thermal decomposition, oxidation, and combustion properties of cotton stalk and sugarcane bagasse powders of different particle sizes. The results showed that all samples exhibited a two-stage reaction mechanism between 200 and 1000 °C, highlighting distinct phases of volatile oxidation and char combustion during thermal breakdown and oxidation [27].

Figure 2b shows the TGA-DTG curves of the individual plastics, representative of those found in RDF. Pyrolysis started at the temperature of 365 °C for polystyrene, 380 °C for polypropylene and polyethylene terephthalate, and 400 °C for polyethylene. Pyrolysis ended for all plastic samples at around 500 °C except for polyethylene at 520 °C. In these temperature ranges, there is weight loss of around 97% for all plastic types except a weight loss of 80% for polyethylene terephthalate. The highest rate of decomposition of plastics occurred at the temperatures of 430, 450, 480, and 490 °C with weight loss rates of 2.9, 2.2, 2.8, and 2.9%/°C, respectively for polystyrene, polyethylene terephthalate, polypropylene, and polyethylene. These individual plastics start to decompose at higher temperatures in comparison with biomass, which is due to the higher thermal stability of plastics. The devolatilization process of both LDPE and HDPE is nearly identical, with only a minor difference observed in the peak heights. This can be attributed to the weak-link model, which suggests that the bonds in the side branches of HDPE break at higher rates. Consequently, their breakage initiates the reaction and results in a lower peak height [28].

Polystyrene: The thermal degradation of polystyrene involves a complex mechanism of chain scission and depolymerization. TG/DTG analysis shows a single mass loss phase. The degradation process follows first-order kinetics [29]. It begins with chain-end scission, yielding styrene as the main product [30]. A radical chain mechanism is proposed to describe the degradation pattern [31]. The composition of volatile products is largely dependent on the degree of conversion but remains relatively unaffected by temperature [32]. The ratio of chain scission to depolymerization remains consistent across different molecular weights and temperatures, enabling the creation of a master curve linking molecular weight reduction to volatile mass [31].

Polyethylene terephthalate: The thermal degradation mechanism of polyethylene terephthalate has been extensively explored using various analytical methods. TG-MS, TG-IR, and Py–GC–MS analyses show that PET degradation begins with homolytic cleavage, followed by β-cis-elimination, yielding benzoic acid and 4-carboxybenzaldehyde as the primary products [33]. Density functional theory calculations indicate that concerted reactions involving six-membered cyclic transition states are energetically more favorable than radical reactions and four-membered cyclic transitions. The main degradation products include terephthalic acid, vinyl terephthalate, and acetaldehyde [34]. Secondary reactions involve the hydrolysis of vinyl esters and dehydration to form olefins, catalyzed by carboxylic acids [35].

Polypropylene: A significant number of studies have explored the mechanism behind the thermal degradation of polypropylene, which suggests that degradation produces alkanes, alkenes, and alkadienes through carbon–carbon bond scission, followed by intramolecular hydrogen transfer. These products form as a result of a free radical mechanism involving primary, secondary, or tertiary radicals, with secondary radicals believed to yield the most degradation products [36]. Under pyrolysis, the breakage of the C-H bond at a tertiary carbon generates stable tertiary radicals. The polymer chain becomes susceptible to cleavage, producing an allyl radical and a secondary radical.

Polyethylene: The thermal degradation mechanism of polyethylene in TG/DTG analysis consists of several essential steps. It begins with the cleavage of the main polymer chain, creating chain-end radicals. Next, both intra- and intermolecular hydrogen transfers take place, leading to the formation of internal radicals and volatile by-products. The final stage is β-scission, which produces volatile compounds and terminally unsaturated polymers [37]. Degradation generally happens in a single step [38], and the process is marked by random chain scission, resulting in a diverse mixture of products that gradually evolve during pyrolysis. Alkanes are predominant in the early phases, while alkenes and trace amounts of alkynes appear later [39].

RDF and mixed biomass/plastics: Fig. 2c shows the TGA-DTG curves of RDF and the prepared mixtures of biomass and plastic. RDF started to decompose at around 200 °C. There are two distinct peaks in the DTG thermogram of RDF confirming the presence of biomass and plastic polymers. The first peak is from 203 °C to 409 °C with a peak of degradation at a temperature of 344 °C and weight loss rate of 0.6%/°C. During this period, the biomass components of RDF start to decompose into light volatiles, which can also be confirmed by the peak degradation temperature of newspaper, cardboard, and wood. The second distinct peak starts from 409 °C to 517 °C with a temperature peak of 486 °C and weight loss rate of 0.36%/°C. This peak shows the decomposition of plastics in RDF. The decomposition of RDF ends at around 520 °C. About 70% weight loss occurred for RDF from 203 °C to 517 °C. The mixture which was a combination of newspaper, cardboard, and wood (biomass) almost had the same decomposition start and end temperature as individual biomasses. It also contained one peak and its peak temperature and weight loss rate were the same peak temperatures and weight loss rates of wood, newspaper, and cardboard. The mixture which involved only plastics, including HDPE, LDPE, PP, PS, and PET showed a similar trend to individual plastics in terms of pyrolysis start temperature and pyrolysis end temperature. It showed one peak and its peak temperature is almost the average peak temperatures of HDPE, LDPE, PP, PS, and PET with values of 462 °C and 1.65%/°C. Mixtures which were composed of different biomasses and plastics, had similar values to RDF in terms of pyrolysis start temperature, pyrolysis end temperature, number of peaks and temperature of peaks. The interaction between biomass and plastic can be attributed to the overlapping release of volatiles from biomass and plastic, leading to volatile–volatile interaction of the pyrolysis gases and the interaction between plastic volatiles and char from the biomass [40]. As lignin and plastics have similar decomposition ranges, the synergistic effect is more obvious in the lignin fraction of the biomass than in the other components of biomass [41]. The DTG of the mixture with 25% wt. plastic and 75% wt. had the most consistency with RDF DTG curve showing that RDF components was mainly biomass and a small proportion of plastic.

## Catalyst Characterization

**Fig. 3:**
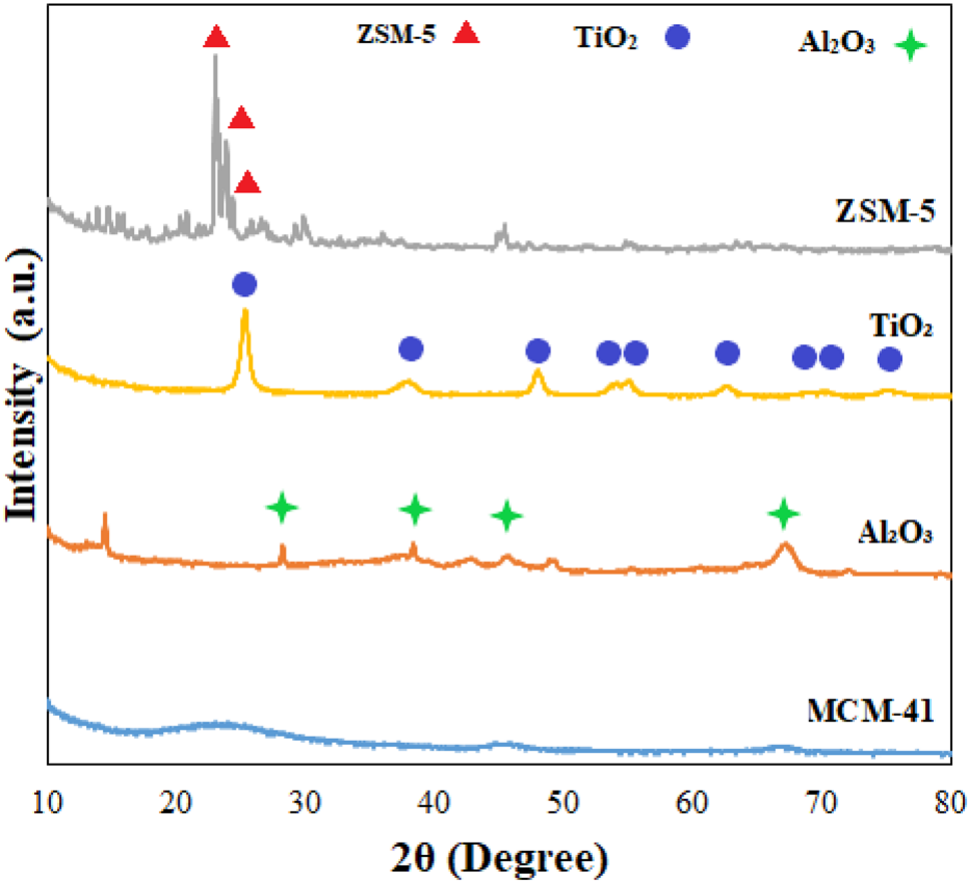
X-ray diffraction of ZSM-5, TiO₂, Al₂O₃, and MCM-41 catalysts

X-ray diffraction (XRD) analysis was undertaken to determine the degree of crystallinity in the investigated catalysts by depicting the sharpness of particular peaks in the derived XRD spectra [42]. Figure 3 illustrates the XRD spectra of the different catalysts used in the experiments, comprising ZSM-5, TiO₂, Al₂O₃, and MCM-41. The XRD pattern of ZSM-5 shows the formation of three main peaks at 2θ of 23°, 23.86°, and 24.35°. The resultant pattern displays identical distinctive peaks observed in ZSM-5 reported in the literature which showed spectral peaks at 23.27°, 23.49° and 24.13° [42, 43]. The XRD spectra of MCM-41 indicated a very mild peak in the range of 2θ between 20° to 30°. This pattern matched the distinctive MCM-41 peaks which have also been observed in the literature [44]. The main Al₂O₃ peaks in the XRD spectra of the Al₂O₃ catalyst were observed at 2θ of 28.3°, 38.2°, 45.8°, and 67.2°. Li et al. [45] carried out the X-ray diffraction of Al₂O₃ in the range of 2θ between 20°-80° and reported the characteristic 2θ peaks of alumina support at, 37.4°, 45.7° and 66.8°, respectively. X-ray diffraction of titanium oxide showed sharp diffraction peaks attributable to anatase, rutile and mixed phases of TiO₂ with 2θ of 25.3°, 37.9°, 48.1°, 54.5°, 54.8°, 62.8°, 69.2°, 70.8°, and 75.5°. The diffraction peaks observed will align with the characteristic patterns of pure TiO₂ phases, as defined by the standard (JCPDS No. 01–075-2546). Nasiri et al. [46] detected diffraction peaks at 2θof 25.25°, 37.77°, 47.96°, 54.01°, 55.01°, 62.61°, 68.80°, 70.21°, and 75.11° correspond to the crystallographic planes (101), (004), (200), (105), (211), (204), (116), (220), and (215), respectively, which are characteristic of the anatase phase of TiO₂ [46].

## Pyrolysis/Non-thermal Plasma/Catalysis of RDF: Product Yield

### Pyrolysis/Non-thermal Plasma

Table 2 shows the yields of gas, oil, and char produced during pyrolysis, pyrolysis/non-thermal-plasma of the RDF at 30, 50, 70 W plasma input power and pyrolysis/non-thermal-plasma catalysis at 70 W using MCM-41, Al₂O₃, ZSM-5, and TiO₂ as the catalyst placed in the plasma reactor discharge gap.

**Table 2:** Product yield (% wt.) for pyrolysis/non-thermal plasma-catalysis of RDF

Input Power (W)	Pyrolysis	30	50	70	70	70	70	70
Catalyst	–	–	–	–	MCM-41	Al₂O₃	ZSM-5	TiO₂
Gas	12.00	12.96	14.93	16.32	23.32	13.76	22.09	14.29
Oil	58.00	57.04	55.07	53.68	46.68	56.24	47.91	55.71
Char	30	30	30	30	30	30	30	30

The results show that 12 wt% gas, 58 wt% oil, and 30 wt% char were obtained during the pyrolysis reaction, that is, in the absence of catalyst or non-thermal plasma. Efika et al. [8] reported on the pyrolysis of RDF at 800 °C at different heating rates in a horizontal tubular reactor, and reported formation of 15.4 wt% gas, 53 wt% oil, and 24.1 wt% solid. Similarly, Williams et al. [47] pyrolyzed RDF in a fixed bed batch reactor and produced 19 wt% gas, 49 wt% oil, 35 wt% char. Table 2 shows that as the input power was increased from 30 to 50 W and then to 70 W, the gas yield showed an increasing trend while the oil yield decreased. Raising the input power from 30 to 70 W resulted in 3.36 wt.% increase in gas yield from 12.96 wt% to 16.32 wt% and a corresponding decrease in oil yield from 57.04 wt% to 53.68 wt%. The char yield did not change because it was held in the first stage reactor and no interaction with the plasma parameters occurred in the pyrolysis reactor. In the reactor system used here, the initial stage involves pyrolysis volatile production. These volatiles then enter the non-thermal plasma reactor. Within this reactor, the feedstock molecules collide with plasma generated energetic species, causing chemical bonds to break and leading to the decomposition of the molecules into ions, atoms, and free radicals. These free radicals can later recombine, forming oil and gas products [48]. In addition, increasing the input power can boost both electron density and gas temperature. As a result, a higher conversion rate is anticipated due to the increased number of energetic electrons [49].

Refuse-derived fuel (RDF) is largely composed of biomass components and different types of thermoplastics, therefore, it is interesting to compare the results produced here with reported literature on plastics and biomass, involving pyrolysis and non-thermal plasma processing. In our previous work, it was observed that increasing the input power to 70 W resulted in more gas and lower liquid production during pyrolysis/non-thermal-plasma processing of polystyrene, biomass, and their mixture. The increase in input power led to the production of more gas, confirming that the pyrolysis vapors were cracked into gases within the non-thermal plasma [13]. In our second study [48], furfural, an oxygenated bio-oil model compound, was upgraded through deoxygenation using a non-thermal plasma process, with hexadecane serving as a hydrogen donor. It was observed that in the absence of plasma, no gaseous products were produced. A direct relation was observed between raising the power from 0 to 70 W and higher gas and oil production. The highest gas and liquid yields, 10.30 wt% and 11.32% wt% respectively, were obtained at 70 W [48]. Aminu et al. [50] worked on pyrolysis/non-thermal plasma catalytic cracking of individual thermoplastics and reported that higher input plasma powers resulted in increased gas production, affirming the conversion of pyrolysis vapours into gases within the non-thermal plasma environment. Meng et al. [52] employed a dielectric barrier discharge non-thermal plasma reactor to degrade tar generated in a fluidized bed gasification reactor. They observed that increasing the applied voltage resulted in a higher specific energy density within the DBD reactor. This increase elevated the energy of electrons within the discharge space of the reactor, thereby significantly improving the probability of high-energy electron collisions with tar molecules. Consequently, this enhanced the efficiency of tar removal. Nguyen and co-authors [53] investigated the conversion of high-density polyethylene into hydrogen and light hydrocarbons using non-thermal plasma. They observed that increasing the plasma power from 10 to 60W resulted in an increase in total gas yield. The rise in plasma power led to a steady increase in the rate of gaseous product formation (Table 2). This relationship can be explained by the higher concentration of plasma-active species, which in turn accelerates the reaction kinetics. Blanquet et al. [54] reported a 21% reduction in hydrocarbon tar content using a pyrolysis non-thermal plasma process compared to a pyrolysis-catalysis process for the catalytic steam reforming of biomass. They also demonstrated that plasma processing of biomass significantly increased the overall gas production from pyrolysis volatiles, compared to catalytic steam reforming without plasma. Gao et al. [55] reviewed the use of DBD plasma-assisted catalytic dry reforming of methane. They found that increasing the input power in a DBD plasma reactor led to higher conversion rates of CH_4_ and CO₂. This effect is due to the fact that higher input power boosts electron density, which speeds up collisions between reaction gas molecules and high-energy electrons. As a result, reactants are activated more effectively. The excited, dissociated, and ionized molecules then drive the dry reforming reaction of methane. Liu et al. [56] investigated toluene reforming in a non-thermal plasma system and found that increasing the input plasma power from 39 to 90 W improved the conversion efficiency of toluene. They attributed this improvement to the increased number of microdischarges resulting from higher discharge power. This boost in microdischarges creates more reaction channels and reactive species during the reforming process, thereby enhancing the overall conversion of toluene. Taghvaei et al. [57] found that increasing the voltage improved both discharge power and guaiacol conversion during its hydrodeoxygenation in a DBD non-thermal plasma reactor. Higher voltages generate a stronger electric field and more intense microdischarges, which in turn increase the energy and electron density in the discharge zone. This elevated energy level enhances the likelihood of electron impact dissociation reactions, including ionization, excitation, and dissociation of gas molecules. Consequently, the increased number and effectiveness of collisions with reactive species lead to a higher probability of the breaking of guaiacol chemical bonds.

Increasing the input plasma power affects the reaction temperature within the plasma discharge area [58]. Xu et al. [58] studied how plasma temperature impacts gaseous products across a range from ambient temperature to 500 °C, with a discharge power of 15W and steam flow rate of 6 mL/h/g biomass, without using a catalyst. They found that the highest yields of all gaseous products, including H₂, occurred at 200 °C. In non-thermal plasma conditions without additional heating, the greatest hydrogen and overall gas yields were observed after 200 °C, likely due to the self-heating effect of the plasma discharge. This effect can elevate the reactor temperature from 100 to several hundred degrees, depending on the energy input. Wang et al. [59] investigated biomass pyrolysis combined with non-thermal plasma reforming to produce hydrogen. They explored various reforming temperatures within a non-thermal plasma reactor, ranging from 250 to 550 °C with an input power of 15 W. They observed that the highest yield of H₂ was achieved at 250 °C. Lower temperatures are preferred to enhance the effectiveness of the non-thermal plasma in the reforming process. This preference is due to the fact that at higher temperatures, the mean electric fields across the discharge gap decrease, which suggests a reduction in mean electron energy density. Consequently, these lower-energy electrons are less effective at breaking the molecular bonds of the pyrolysis volatiles.

## Pyrolysis/Non-thermal Plasma Catalysis

The introduction of the MCM-41 and ZSM-5 catalysts to the non-thermal plasma process at 70 W input power led to more gas production (Table 2), at 23.32 wt% for MCM-41 and 22.09 wt% for ZSM-5, in comparison to the absence of catalyst at 70 W. There was a corresponding decrease in the oil yield in the presence of MCM-41 and ZSM-5, respectively at 70 W. The presence of Al₂O₃ and TiO₂ as the catalyst in the non-thermal plasma reactor at the input power of 70 W promoted production of oil when compared to pyrolysis/non-thermal plasma processing at 70 W with a corresponding lowering of the gas yields. The high surface areas of MCM-41 (734 m^2^ g^−1^) and ZSM-5 (233 m^2^ g^−1^) played a significant role in effectively breaking down high-molecular-weight pyrolysis volatiles into lower-molecular-weight hydrocarbons. These hydrocarbons were subsequently reformed into syngas. Aminu et al. [60] carried out pyrolysis/non-thermal plasma/catalytic steam reforming of HDPE in the presence of solid acid support materials. It was suggested that the combination of plasma with MCM-41 and Y-zeolite led to a more intensified electric field, which effectively cracked more of the HDPE pyrolysis volatiles, resulting in increased gas production. Blanquet et al. [54] investigated the processing of biomass using pyrolysis-catalysis, pyrolysis-plasma, and pyrolysis-plasma-catalysis with Ni/Al₂O₃ as the catalyst and the input power of 40 W in the presence of steam. They reported that gas yield was slightly higher in the presence of both plasma and catalyst compared with only plasma. Fan et al. [14] investigated plasma-catalytic upgrading of biomass pyrolysis volatiles to bio-fuel at the reaction temperature of 400 °C, system pressure of 5 kPa and the discharge power of 25 W. They reported formation of 19.5% char, 44.95% gas, and 35.55% liquid in the presence of HZSM-5 and non-thermal-plasma, which was 2.83% higher than gas yield and 2.87 lower than liquid yield in catalysis alone. They mentioned that once non-thermal plasma and catalyst were introduced, the cracking performance was greatly improved, the yield of oil declined and the yield of gas clearly increased. They concluded that the reduction in the proportion of the organic phase was clearly a trend, indicating that increased cracking caused more organic matter to engage in catalytic reactions. During these reactions, some oxygen was removed as H₂O. Although some hydrogen was also removed, it can be inferred that a greater amount of oxygen was eliminated as COx. This suggests that the oxygen content was significantly reduced, which implies that the fuel grade should be further improved. Xu et al. [58] examined the impact of plasma and plasma-catalysis on tar reduction at a reforming temperature of 500 °C, with a discharge power of 15 W, a steam velocity of 6 mL/h/g biomass, and using a Ni–Fe/–Al₂O₃ catalyst. They found that applying plasma discharge resulted in a moderate decrease in tar yield compared to thermal heating alone at 500 °C. This reduction was due to the enhanced effects of the non-thermal plasma in conjunction with the temperature, which improved tar removal efficiency. The most effective tar elimination was achieved with the combined plasma-catalysis system, which benefited from the synergistic effects of the bimetallic catalyst and non-thermal plasma, particularly through thermal cracking and reforming processes. Fan et al. [61] used the concept of non-thermal plasma synergistic catalysis for enhancing the conversion of biomass pyrolysis vapours to produce biofuels. They reported that the plasma-catalysis system with HZSM-5, Ti- HZSM-5, and Zn- HZSM-5 as the catalysts showed increase in gas product yields while simultaneously reducing the yields of liquid and bio-oil in comparison to the catalysis system. They concluded that plasma-catalysis enhances product yields by significantly improving catalytic cracking performance and reaction efficiency. The introduction of non-thermal plasma, especially with Ti/HZSM-5, activates and breaks down complex organic molecules more effectively, resulting in increased gas-phase product yields. This process also reduces the formation of liquid and bio-oil, as the plasma technology promotes the conversion of larger molecules into smaller hydrocarbons and hydrogen, which contribute to the higher gas yields. Additionally, plasma-catalysis lowers the oxygen content of bio-oil, further refining its physicochemical properties. Fan et al. [21] utilized a dielectric barrier discharge non-thermal plasma to aid in the conversion of biomass into aromatic hydrocarbons under vacuum pyrolysis and zeolite catalysis conditions. The utilization of metal modified HZSM-5 catalysts compared to HZSM-5 improved the reforming performance of the volatiles, resulting in a reduction in liquid yield and a noticeable increase in gas yield. Xiao et al. [51] conducted polypropylene pyrolysis over zeolite ZSM-5 in a two-stage fixed-bed pyrolysis system incorporating a coaxial dielectric barrier discharge (DBD) plasma reactor. Increasing plasma power during the plasma-catalysis pyrolysis of polypropylene affects the yields of gas, oil, and wax significantly. Initially, introducing plasma at 60 W increases the gas yield as it facilitates the cleavage of C–H and C–C bonds, leading to the formation of lighter hydrocarbons and gases such as H₂ and CH₄, while reducing the wax yield. As plasma power is further increased, the gas yield starts to decrease due to the potential breakdown of lighter hydrocarbons or side reactions. The oil yield remains relatively stable throughout the process, as plasma predominantly impacts heavier hydrocarbons rather than the oil fractions. The wax yield decreases substantially with increasing plasma power, reaching near elimination at 120 W, as plasma enhances the cracking of heavy hydrocarbons into lighter gases and products. They suggested that plasma modification could potentially enhance the catalytic activity of ZSM-5, with the stronger catalytic activity of ZSM-5 being particularly advantageous for promoting oil production. Chang et al. [62] carried out non-thermal plasma and non-thermal plasma-catalysis for toluene removal using Mn/N-doped carbon as catalyst. They observed that a higher specific energy density (SED) resulted in significantly improved toluene removal efficiency, regardless of whether a catalyst was used; this was because higher SED produced more high-energy electrons and active species in the discharge region, leading to greater efficiency. However, as the SED increased, the energy yield dropped notably because more energy was spent on converting intermediates or exciting the carrier gas than on removing toluene. Additionally, the NTP-catalyst system achieved much higher efficiency and energy yield. The non-catalyzed NTP system removed 90% of the toluene at a relatively high SED value (210 J⋅L⁻^1^), while the SED values for the NTP-catalyst systems ranged from 150 to 185 J⋅L⁻^1^. The NTP-6% Mn/N–C system achieved 98% toluene removal efficiency at an SED value of 252 J⋅L⁻^1^. Xiao et al. [63] compared plasma-catalytic pyrolysis with traditional catalytic pyrolysis. Initially, pyrolysis of polypropylene without plasma at 650 °C produced a high amount of liquid (62 wt%) and some gas (38 wt%), with no solid byproduct. As the catalytic temperature increased, the yield of liquid decreased while the gas yield increased, stabilizing at about 50 wt% for both. When plasma was introduced, the gas yield increased and the liquid yield decreased more significantly as the temperature rose. At 800 °C, the gas yield peaked at 56 wt% and the liquid yield fell to 44 wt%. This indicated that plasma enhances the conversion of liquids to gases similarly to higher temperatures.

## Pyrolysis/Non-thermal Plasma/Catalysis of RDF: Gas Composition

### Pyrolysis/Non-thermal Plasma

**Fig. 4:**
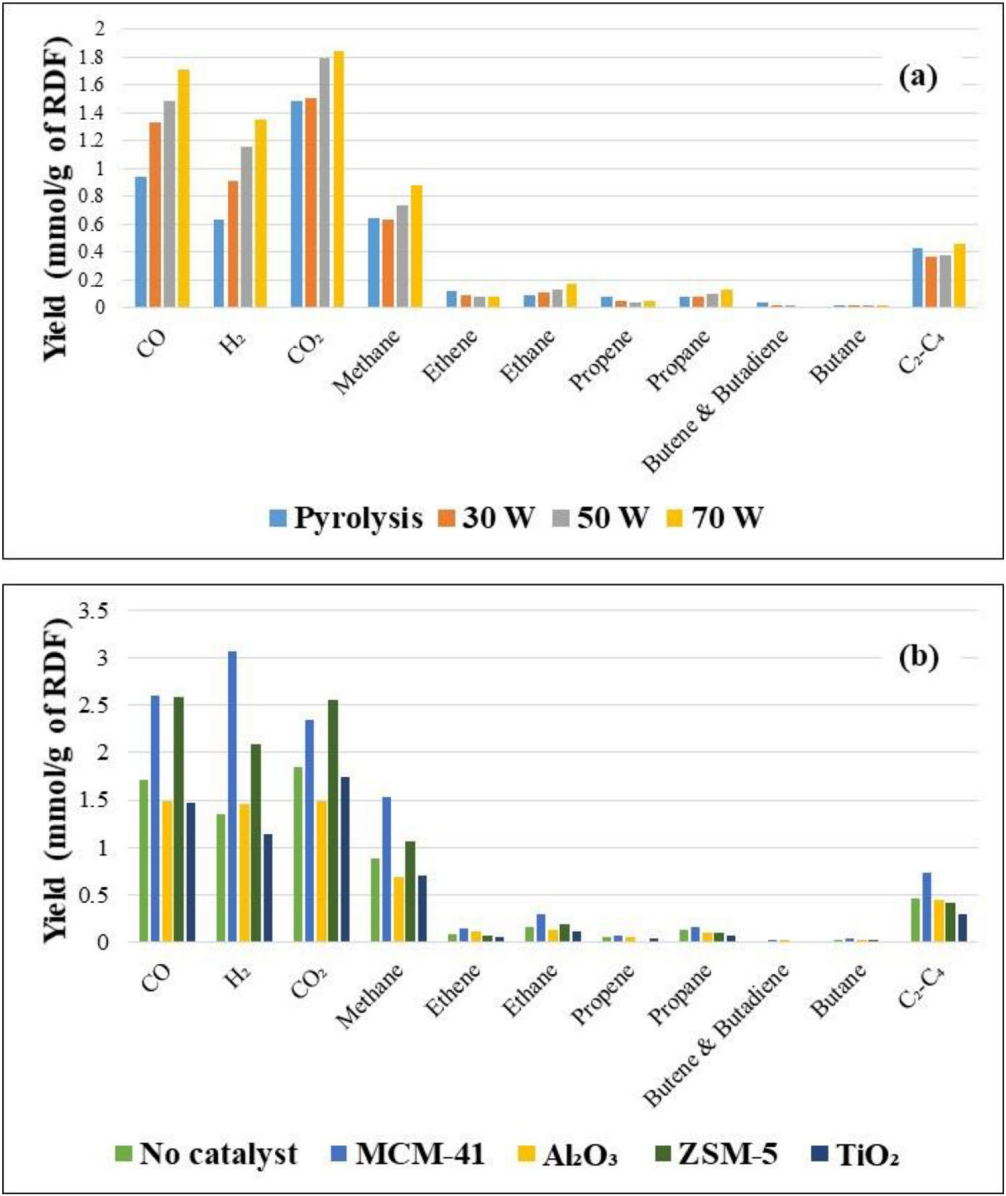
**a** Gas composition (mmol/g of feedstock) in pyrolysis/non-thermal plasma processing of RDF at 0, 30, 50, and 70 W 5 **b** Gas composition (mmol/g of feedstock) from the pyrolysis/non-thermal plasma-catalysis processing of RDF at 70 W with MCM-41, Al₂O₃, ZSM-5, and TiO₂

The yield and composition of the gas produced from the processing of RDF was studied under pyrolysis, pyrolysis/non-thermal-plasma at 30, 50 and 70 W and pyrolysis/non-thermal-plasma-catalysis at 70 W in the presence of different catalysts, including MCM-41, Al₂O₃, ZSM-5, and TiO₂. Figure 4a shows the gas components yields (mmol/g of feedstock) from the pyrolysis and the pyrolysis/non-thermal plasma processing of RDF at the input powers of 30 W, 50W, and 70W. The product gases were mainly composed of CO, CO₂, H₂ and CH_4_ along with lower concentrations of C_2_ – C_4_ hydrocarbons. In non-thermal plasma reactors dealing with hydrocarbons, the initial stage of the process involves generating energetic electrons, usually with an average energy of 5 eV in DBD reactors [64]. Some of these electrons, having sufficient energy, collide with hydrocarbon molecules, leading to the breaking of C–C and C–H bonds, which generally require about 3 eV of energy. These collisions between electrons and hydrocarbons produce small activated ions and radicals, which can later recombine to form hydrogen and lighter hydrocarbons such as alkanes and olefins [64].

The introduction of the plasma increased the yield of CO, H₂, CO₂, and alkanes, including methane, ethane, propane, and butane and were further increased with increasing input plasma power. Decarboxylation and decarbonylation of the oxygenated hydrocarbons during the non-thermal plasma process are two possible deoxygenation reactions of the RDF-derived pyrolysis volatiles, as indicated by the increased production of CO and CO₂ at increasing input powers. For example, CO₂ with the yield of 1.49 mmol g^−1^ in pyrolysis increased gradually to 1.85 mmol g^−1^ at 70 W and CO yield was raised from 0.94 mmol g^−1^ in pyrolysis to 1.71 mmol g^−1^ at 70 W. The production of hydrogen for potential hydrodeoxygenation reactions is indicated by higher yields of hydrogen in the plasma system which were increased as plasma input power was raised. The results are consistent with our two previous studies, which showed most gases yields (CO, CO₂, H₂, and CH_4_, etc.) increased with increased input plasma power during the non-thermal plasma processing of both polystyrene-biomass pyrolysis volatiles and furfural-hexadecane vapors [13, 48]. Nguyen et al. [53] worked on decomposing high-density polyethylene into hydrogen and light hydrocarbons and observed that the overall gas production increased as the input power was increased, particularly for hydrogen and methane. An increase in hydrogen yield at higher input powers was attributed to the endothermic cracking of long-chain C-H fragments, a process facilitated by the elevated temperatures observed at high plasma power levels. Others have reported that increased input plasma power produces higher yields of gas [53, 54, 58, 59, 65, 66], which were mainly attributed to the higher abundance of electrons generated through intensified micro-discharges in the non-thermal plasma/catalytic process. This increase in electrons provided additional reaction channels and reactive species, thereby facilitating more reaction processes. Fridman reported [67] electrons generated in a non-thermal plasma possess more energy than the dissociation energies of C − H and C − C chemical bonds.

## Pyrolysis/Non-thermal Plasma Catalysis

Figure 4b shows the yields of gas compounds produced from pyrolysis/non-thermal-plasma processing at an input plasma power of 70 W and pyrolysis/non-thermal-plasma-catalysis at 70 W and catalysis with MCM-41, Al₂O₃, ZSM-5, and TiO₂_._ Introduction of MCM-41 and also ZSM-5 to the non-thermal plasma system produced high amounts of CO and CO₂ suggesting that MCM-41 and ZSM-5 were effective in the deoxygenation of the RDF derived pyrolysis volatiles in the presence of non-thermal-plasma. In terms of CO and CO₂ yield as an indication of deoxygenation of the RDF pyrolysis volatiles, the order of effectiveness of the four catalysts investigated was, MCM-41 and ZSM-5 > Al₂O₃ and TiO₂_._ The results suggest that the MCM-41 and ZSM-5 catalysts combined with the non-thermal plasma environment leading to synergistic effects, resulting in increased total gas yield, hydrogen production and deoxygenation reactions. The differences between the MCM-41 and ZSM-5 compared with the Al₂O₃ and TiO₂ catalysts can be attributed in part to differences in discharge characteristics when these materials are loaded in the discharge region. In addition, surface area, porosity, and dielectric constant of the catalysts play significant roles in influencing plasma-catalyst synergy [23, 60].

In terms of catalyst surface area in plasma-catalysis, the discharge transitions from a filamentary discharge to a mix of microdischarges and surface discharges on the catalyst surface occur, with the majority of the discharge occurring on the catalyst’s surface. Consequently, a catalyst with a larger surface area generates more surface discharges, leading to increased tar cracking [60]. Patil et al. [68] highlighted that the conventional mechanism is less significant in plasma-catalysis, with the primary contribution coming from the generation of micro-discharges. It is suggested that porous catalyst support materials within the plasma zone create a stronger electric field around the pores. This field affects the creation of plasma species and triggers micro discharges within the pores [23]. It has also been noted that the environment of plasma and catalysts, which includes reactive gases, can produce plasma discharge streamers with high electron densities. These streamers might be capable of penetrating nanometer-sized pores in the catalyst [69]. Dielectric constant measures the potential electrical energy stored in a material when an electric field is applied. In plasma catalysis, the dielectric constant of the catalyst’s support material determines the strength of the electric field, affecting the extent of plasma enhancement within the catalyst’s pores [23]. MCM-41 and ZSM-5 have low dielectric constants which could be due to their mesoporous structures [70, 71]. The dielectric constant of ZSM-5 zeolite varies depending on its structure and composition. Low-k dielectric materials derived from ZSM-5 exhibit dielectric constants ranging from 2.27 to 2.78, with the value increasing as the structure evolves from zeolite to SiO₂-Al₂O₃ glass and finally to SiO₂-based cristobalite ceramics [71]. The dielectric properties of ZSM-5 are influenced by temperature and ammonia storage, with the complex dielectric permittivity closely correlating with the stored ammonia mass [72]. The dielectric constant of Al₂O₃ has been consistently reported to be around 8–9 at room temperature. Govinda et al. [73] measured a value of 9.4 for single crystals of Al₂O₃ at 30 °C, which was independent of frequency. Ion et al. [74] found a slightly lower value of 8.3 at 273 K for thin films deposited by pulsed laser deposition. This value aligns with Hickmott’s findings of ~ 8.3 at 295 K for anodized Al₂O₃ films [75]. The dielectric constant of TiO₂ varies depending on its crystal structure and processing conditions. For rutile TiO₂, values range from 160 to 240 along the c-axis [76]. In contrast, anatase TiO₂ films show lower dielectric constants of approximately 40 [77, 78]. It can be concluded that the catalysts with lower dielectric constant, including MCM-41 and ZSM-5 show greater activity such as, higher H₂ production and more deoxygenation (CO and CO₂ production) compared to Al₂O₃ and TiO₂ catalysts with higher dielectric constant. It is due to the fact that materials with lower dielectric constants facilitate micro-discharges within catalyst supports more readily than materials with higher dielectric constants like titanium oxide [23]. Patil et al. [68] proposed that when an electric field is applied to a material with a high dielectric constant, the material easily becomes polarized. This polarization generates an internal electric field that opposes the overall applied electric field, leading to a reduction in electron energy. Consequently, this results in lower energy of reactive species and decreased gas ionization.

The results suggest that the incorporation of the catalysts in the plasma regime, particularly the MCM-41 and ZSM-5 catalysts improves product selectivity and yield. This indicates that the catalyst effectively utilizes the active species produced by the plasma. The active surface provided by the catalysts facilitates the interaction of excited reactive species, leading to more efficient product formation [53]. Moreover, the non-equilibrium nature of the plasma discharge promotes the adsorption of active species on the catalyst surface, thereby increasing the likelihood of chemical reactions. Solid acid catalysts such as MCM-41 and ZSM-5 are recognized for their role as proton-donor acidic catalysts and promote cracking reactions due to their sinusoidal pore structure.

Xiao et al. [51] researched polypropylene pyrolysis over zeolite ZSM-5 in a two-stage fixed-bed pyrolysis system, incorporating a coaxial dielectric barrier discharge (DBD) plasma reactor. They reported that the quantities of H₂, CH_4_, C_2_H_6_, C_2_H_4_, and C_3_H_8_ exhibited a remarkable increase compared to the plasma-alone and catalyst-alone modes, indicating a significant synergistic effect between the plasma and catalyst. Plasma catalysis created a more reactive environment with a higher concentration of energetic species, promoting the formation of carbocations and radicals within the hydrocarbon chains. This facilitated the cleavage of C–C and C–H bonds, thereby enhancing the production of light hydrocarbons and H₂. Simultaneously, exposure of the catalyst to the plasma altered its morphology, acidic sites, and oxidation state, resulting in increased catalyst activity. Xu et al. [58] investigated integrated pyrolysis and plasma-catalysis system using a bimetallic Ni–Fe/γ-Al_2_O_3_ catalyst in the plasma reforming stage. The results showed that packing the bimetallic catalyst into the plasma zone significantly enhances gas yields, including H_2_ and CO, at all temperatures. At 500 °C, the plasma-catalysis system achieved optimal H_2_ (47.65 mmol/g) and total gas yields (70.53 mmol/g), significantly outperforming both plasma-only and catalyst-only modes. The synergy between plasma and catalyst was most pronounced at higher temperatures, where catalyst activation and plasma interaction combined to enhance overall performance. Although plasma reforming works well at lower temperatures, the catalyst becomes the dominant factor in improving performance at due to activation higher temperatures. Aminu et al. [60] carried out pyrolysis-non-thermal plasma reforming of waste HDPE using different catalyst support materials. The highest hydrogen yield of 11 mmol g^−1^_plastic_ was achieved using MCM-41 as the catalyst, with Y-zeolite producing a yield of 9.1 g^−1^_plastic_. They attributed the varying performances of the different packing materials to their distinct discharge characteristics when placed in the discharge region. They observed that although MCM-41, Y-zeolite, and ZSM-5 catalysts share the same chemical composition, the hydrogen production in the presence of these catalysts was dependant to their surface area.

They also mentioned that materials with higher dielectric constants, such as BaTiO_3_ and CaTiO_3_, exhibited poorer performance in terms of hydrogen yield during plasma-catalytic reforming reactions. The interaction of non-thermal plasma with these high dielectric materials led to a reduced effective electric field and lower electron energy, which was insufficient to crack most of the derived pyrolysis hydrocarbons. The increased production of hydrogen from the decomposition of the plastic polypropylene in the non-thermal plasm environment creates in situ hydrogen for the hydrodeoxygenation of the bio-oil derived from the biomass fraction of the RDF.

## Pyrolysis/Non-thermal Plasma/Catalysis of RDF: Oil Composition

### Pyrolysis/Non-thermal Plasma

**Fig. 5:**
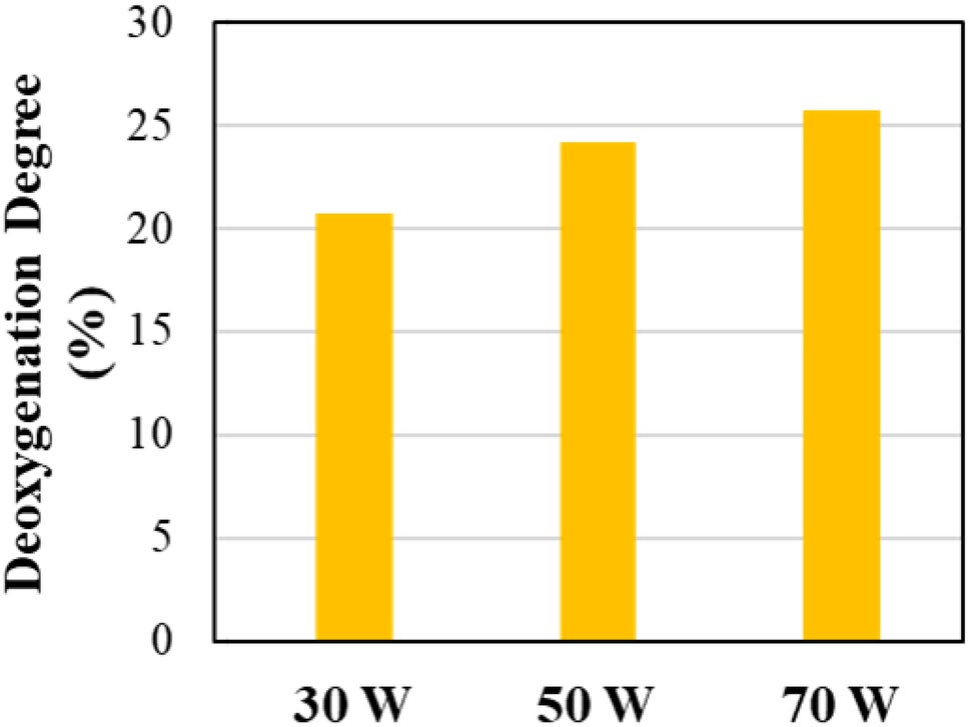
Deoxygenation degree of oil from pyrolysis/non-thermal-plasma processing of RDF at 30, 50, and 70 W

Table 3 shows the compounds present in the oil produced from pyrolysis and pyrolysis/non-thermal plasma of RDF at 30, 50, and 70 W. GC–MS was used to identify the compounds. Figure 5 presents the degree of deoxygenation (%) of the produced oil from the pyrolysis/non-thermal-plasma processing of RDF at different input powers compared to the oil produced from pyrolysis of RDF based on the data in Table 3. The main compounds present in the product oil are oxygenated compounds such as phenol and phenol derivatives, organic acids, aldehydes, ketones and hydroxy compounds. Considering the three main oil compounds at each input power reveals that furans are the most produced compounds in the absence of the plasma. These furans contained one, two or three oxygen atoms in their structure. At the input power of 30 W, furan and phenols are the main compounds, but they still had three or two oxygen atom. Raising the power to 50 W generated furans and phenols with lower oxygen atom content (one and two oxygen). At the input power of 70 W, the main peaks were phenol groups with either one or two oxygen atom. This trend shows that in the absence of plasma or in the low input powers, the main compounds had higher oxygen atoms while in the higher input powers, the number of oxygen atoms in main compounds was lower. The yields of most oxygenated compounds decrease when comparing the results between pyrolysis and pyrolysis/non-thermal plasma at 70 W. There is a direct relation between increasing the input power and a higher degree of deoxygenation. The highest deoxygenation occurred at 70 W at 25.7%. This phenomenon may be explained by the observation that raising the applied voltage leads to an increase in both the quantity and energy levels of electrons and ions, consequently causing the breakdown of stronger chemical bonds [13]. Hosseinzadeh et al. [79] investigated the decomposition of a lignin bio-oil model compound, specifically 4-methylanisole, through utilization of a DBD plasma reactor. They observed selectivity towards 4-methylphenol, 2,4-dimethylphenol, and 1-ethoxy-4-methylbenzene, which exhibited a tendency to decrease as the voltage was elevated. There was a consequent trend for an increase in lighter compounds, mainly mono-aromatic and phenolic compounds such as xylene, phenol, alkylated phenols, toluene and benzene. Taghvaei et al. [57] noted that increasing the voltage led to enhanced discharge power, guaiacol conversion, and deoxygenation during the hydrodeoxygenation of guaiacol in a dielectric barrier discharge (DBD) non-thermal plasma reactor. By applying a plasma discharge power of 100 W, hydrogen was generated in situ, causing the breakdown of methyl and methoxyl radicals derived from lignin pyrolytic oil. This decomposition produced mono-oxygenated compounds such as phenol, methylphenols (including 2-methylphenol and 4-methylphenol), and dimethylphenols (2,4–2,6 and 3,4-dimethylphenol), along with benzene, toluene, and xylene. In addition, small amounts of anisole, catechol, methylanisoles, cyclohexanol, and trimethylphenols were detected. Elevated applied voltages created a stronger electric field and more robust micro-discharges, increasing the energy and electron density in the discharge zone. Consequently, the likelihood of electron impact dissociation reactions, including processes like ionization, excitation, and dissociation of gas molecules, was heightened. This resulted in an increased probability of breaking guaiacol chemical bonds due to the greater number and effectiveness of collisions with reactive species. In subsequent work, Taghvaei et al. [80] observed that increasing the applied voltage led to a rise in the selectivity for phenol, benzene, toluene, and xylene, while products like oxygenated methylphenols and dimethylphenols showed a decline. These patterns were attributed to the enhanced direct demethoxylation of guaiacol due to the stronger electrical field between the electrodes in the discharge zone at higher voltages. Additionally, methyl radicals can form from demethylation and the cleavage of the O–CH_3_ bond. With more electrons and reactive species, as well as increased average electron energy resulting from the higher voltage, the likelihood of decomposing these methyl radicals (which have a higher bond dissociation energy) into CH₂, CH, and H radicals increased. This heightened decomposition reduced the transalkylation reaction, leading to fewer methyl-substituted ring products like methylphenols and dimethylphenols.

**Table 3:** Oil composition (mg/g of feedstock) in pyrolysis/non-thermal plasma processing of RDF at 0, 30, 50, and 70 W

No	Retention time (min)	Compound	MW	Pyrolysis	Pyrolysis-plasma at 30 W	Pyrolysis-plasma at 50 W	Pyrolysis-plasma at 70 W
1	2.692	2-Propanone, 1-hydroxy	74	36.01	38.10	51.27	31.95
2	3.359	2-Butanone, 3-hydroxy	88	8.47	5.70	9.10	8.41
3	5.448	2H-Pyran, 3,4-dihydro-	84	8.59	8.24	18.20	7.54
4	7.210	Furfural	96	143.55	85.78	69.57	36.42
5	9.046	acetic anhydride	102	6.23	6.41	11.24	7.72
6	11.199	Furan,2-ethyl-5-methyl	110	14.03	9.13	11.57	6.94
7	14.558	2-Furancarboxaldehyde, 5-methyl-	110	46.78	30.01	29.38	20.11
8	14.919	Propanoic acid, anhydride	130	7.93	9.01	10.51	9.14
9	15.131	2-Butanone, 1-(acetyloxy)-	130	7.69	9.43	11.09	11.96
10	16.085	Phenol	94	46.12	17.93	58.27	58.62
11	17.156	unknown	-	8.48	0.00	1.34	0.00
12	18.958	1,2-Cyclopentanedione, 3-methyl-	112	21.22	17.17	10.87	8.00
13	21.059	Phenol,2-methyl	108	11.38	14.19	6.22	5.78
14	22.433	Phenol,3-methyl	108	22.39	22.48	12.02	10.93
15	22.729	Mequinol	124	42.20	40.27	196.04	74.35
16	23.864	Maltol	126	40.50	40.27	9.65	5.15
17	27.143	2-Nonen-1- ol, €-	142	12.11	4.59	9.26	8.93
18	27.281	Phenol, 2-methoxy-5-methyl-	138	19.75	8.71	7.54	216.74
19	27.460	1,2-benzenediol	110	5.42	12.94	7.33	3.17
20	28.046	unknown	144	2.80	14.32	5.25	2.94
21	28.653	2-Furancarboxaldehyde, 5-(hydroxymethyl)	126	68.32	175.74	5.03	2.05

**Fig. 6:**
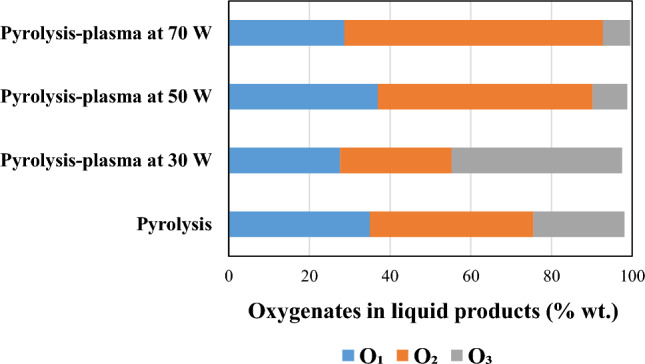
Oxygen atom distribution (% wt.) in the oil products from pyrolysis and pyrolysis/non-thermal plasma processing at 30, 50, and 70 W

Figure 6 shows the compounds distribution based on the number of the oxygen atoms produced from pyrolysis and pyrolysis-DBD plasma processing of RDF at 30, 50, and 70 W input plasma power. The weight percentages were calculated based on sum of the yields of the compounds containing x oxygen atom (x = 1, 2, 3) divided by the total oil yield. The oil from pyrolysis contained 35%, 45.5%, and 22.5% oxygenates with one, two, and three oxygen atoms, respectively. Raising the power to 30 W did not improve the oxygen atom distribution which could be due to the insufficient energy for breaking bonds, but increasing the power to 50 W dramatically decreased oxygenates containing three oxygen atom to 8.6% and a slight increase of 2% in mono-oxygenated compounds compared to pyrolysis. The percentage of oxygenates with three oxygen atoms tended to decrease to 6.7% at 70 W. Double-oxygenated compounds were dominant at 50 W and 70 W with 53.2% and 64%, respectively. Khatibi et al. [48] reported formation of oxygenated and non-oxygenated compounds in the oil produced from non-thermal plasma processing of furfural, an oxygenated bio-oil model compounds at different input powers. They classified the produced oil compounds into three main groups: non-oxygenated compounds, single-oxygen, and dual-oxygen-containing compounds. Toluene, a single-ring aromatic compound, was generated at higher input powers of 50 W and 70 W, showing a direct correlation between increased plasma power and toluene production. Cyclobutene, 2-propenylidene-, another non-oxygenated compound, was produced at plasma powers of 30, 50, and 70 W. The single-oxygenated compounds produced during DBD-plasma processing of furfural include furan, 2-ethyl-5-methyl-, pyrazole-4-carboxaldehyde-1-methyl-, and 2-cyclohexen-1-one. Compared to the feedstock furfural, these compounds have higher hydrogen and lower oxygen content, with their production rates increasing as input power rises. The dual-oxygenated compound, 2-furanmethanol, 5-methyl-, had a yield of 1.87 mg g^−1^ without plasma, but its production decreased by approximately 7.5 times to 0.25 mg g^−1^ at the higher input power of 70 W.

**Fig. 7:**
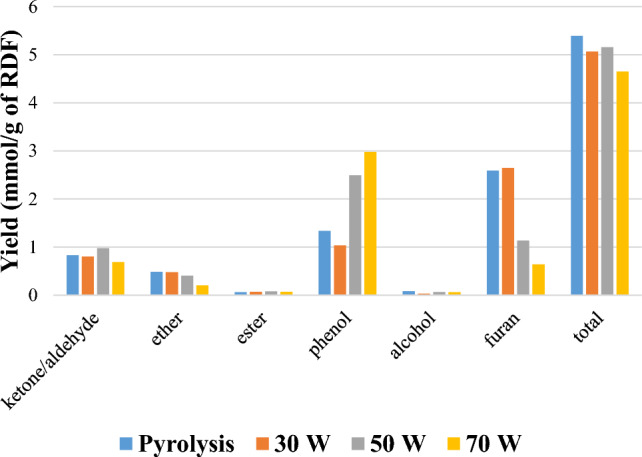
Yield of oxygenated groups (mmol g^−1^ of RDF feedstock) in the oil products from the pyrolysis/non-thermal plasma processing of RDF at 0, 30, 50, and 70 W

Figure 7 classifies the oxygenated groups, including ketone/aldehyde, ether, ester, furan, alcohol and phenol groups present in the oil product from pyrolysis and pyrolysis/non-thermal-plasma processing of RDF based on yield (mmol g^−1^ of feedstock). Organic compounds in the bio-oil primarily comprise acids, aldehydes, ketones, phenols, alcohols, furans, and similar substances [61]. Ketone/aldehyde, ether, furan, and alcohol are the groups which have lower yields in the derived oil from plasma processing at 70 W input power in comparison to pyrolysis. Furan is the main chemical group in pyrolysis and pyrolysis/non-thermal plasma at 30 W while at the input power of 50 and 70 W, phenol is the major group. Production of phenols increased with raising the power to 70 W compared to pyrolysis which shows the formation of other oxygenated groups to phenol. The oxygen atoms present in the phenolic hydroxyl form were difficult to eliminate. This difficulty stemmed from the protective influence of the highly stable benzene ring structure on one hand, and the insufficient contact with the high-cracking catalyst on the other hand [61]. Generally, the yields of oxygenated compounds declined for RDF pyrolysis/non-thermal plasma processing of RDF at 70 W input plasma power compared to pyrolysis. The observed decreases confirm that the deoxygenation process occurred upon applying the plasma. In our previous work [13], a similar trend was observed during pyrolysis/non-thermal plasma processing of biomass where increasing the input power led to lower production of ketones, furans, and phenols. These findings align with research conducted by Taghvaei et al. [57], who explored plasma upgrading of guaiacol through hydrodeoxygenation reaction without directly supplying hydrogen as a feed. The necessary hydrogen was generated in situ through the plasma decomposition of methoxyl and methyl radicals present in the chemical structure of lignin pyrolytic oil at room temperature. By applying discharge plasma power, hydrogen was generated in-situ, leading to the decomposition of methyl and methoxyl radicals originating from lignin pyrolytic oil. A high yield of mono-oxygenated compounds was achieved. Phenol as the main products was selectively produced through direct demethoxylation of guaiacol, involving the direct hydrogenolysis of the C–O bond in guaiacol. Major compounds were mono-oxygenated compounds such as phenol, methylphenols (2-methylphenol and 4-methylphenol), dimethylphenols (2,4–2,6 and 3,4-dimethylphenol), as well as benzene, toluene, and xylene.

## Pyrolysis/Non-thermal Plasma Catalysis

**Fig. 8:**
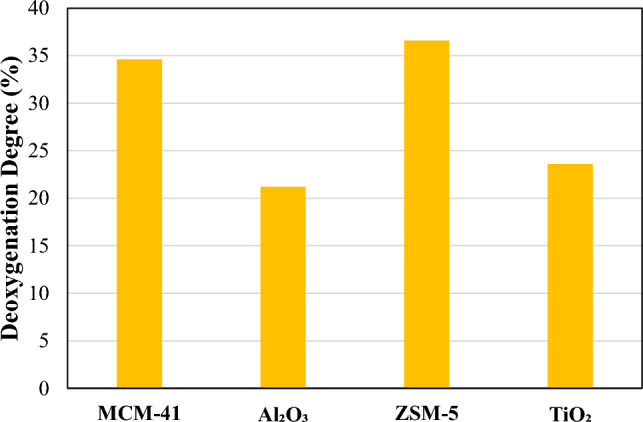
Deoxygenation degree of oil in pyrolysis/non-thermal-plasma-catalysis processing of RDF at 70 W with MCM-41, Al₂O₃, ZSM-5, and TiO₂

The composition of oil products and their yields based on the mass of RDF (mg g^−1^) from pyrolysis and pyrolysis/non-thermal plasma-catalysis of RDF at 70 W in the presence of MCM-41, Al₂O₃_,_ ZSM-5, and TiO₂ catalysts is presented in Table 4. The degree of deoxygenation in the non-thermal-plasma-catalysis processing of RDF at 70 W with different catalysts is shown in Fig. 8. All the main produced compounds detected in Table 4 are oxygenated except for pyridine produced by TiO₂. This could be due to the much higher proportion of biomass which is at least 75% wt. of RDF compared to the plastic percentage (Fig. 2c). No formation of aromatic compounds was also observed, probably due to the operation at low temperature and atmospheric pressure [57].

**Table 4:** Oil composition (mg/g of feedstock) in pyrolysis/non-thermal plasma-catalysis processing of RDF at 70 W with MCM-41, Al₂O₃, ZSM-5, and TiO₂

No	Retention time (min)	Compound	MW	MCM-41	Al₂O₃	ZSM-5	TiO₂
1	2.659	2-Propanone, 1-hydroxy	74	0.00	37.90	12.47	8.86
2	2.939	Butanal, 3-hydroxy-	88	7.10	3.58	0.00	0.00
3	3.186	4-Cyclopentene- 1,3-diol, trans-	100	15.63	6.80	8.75	8.80
4	3.293	2-Butanone, 3-hydroxy	88	0.00	16.91	27.83	10.83
5	3.474	Propanoic acid	74	0.00	13.45	0.00	12.35
6	4.110	Pyridine	79	0.00	0.00	0.00	15.41
7	5.390	2H-Pyran, 3,4-dihydro-	84	13.16	12.11	12.00	25.68
8	7.040	Furfural	96	0.00	97.77	10.37	32.21
10	10.775	Furan, 2,4-dimethyl-	96	3.64	10.73	6.91	28.45
11	11.087	Furan,2-ethyl-5-methyl	110	3.42	17.32	4.78	23.46
12	14.403	2-Furancarboxaldehyde, 5-methyl-	110	0.00	45.34	0.00	0.00
13	14.413	Furan, 2,5-dimethyl	96	7.67	0.00	0.00	0.00
14	14.417	1(1-Butyne)cyclopentanol	138	0.00	0.00	11.75	38.33
15	14.737	Propanoic acid, anhydride	130	0.00	19.85	0.00	5.27
16	14.946	2-Butanone, 1-(acetyloxy)-	130	0.00	20.21	0.00	5.91
17	15.928	Phenol	94	42.72	46.06	215.50	45.77
18	18.707	1,2-Cyclopentanedione, 3-methyl-	112	11.59	13.42	5.83	13.23
19	19.373	2-Cyclopenten-1-one, 2,3-dimethyl-	110	4.85	11.17	4.77	25.39
20	20.930	Phenol,2-methyl	108	12.55	13.71	12.10	21.34
21	21.451	Unknown	–	5.38	11.80	7.70	8.15
22	22.295	Phenol,3-methyl	108	25.55	28.43	22.49	22.99
23	22.599	Mequinol	124	94.40	27.88	16.50	157.33
24	23.721	Maltol	126	4.21	9.96	4.87	0.00
25	24.003	Phenylethyl alcohol	122	3.50	14.04	14.86	8.04
26	26.552	Phenol, 2,5-dimethyl	122	3.50	12.16	14.25	9.81
28	27.205	Phenol, 2-methoxy-5-methyl-	138	25.45	11.43	12.23	8.35
29	27.448	1,2-benzenediol	110	7.26	11.86	13.50	8.29
30	28.019	Unknown	144	171.78	21.49	16.61	6.47
31	28.596	2-Furancarboxaldehyde, 5-(hydroxymethyl)	126	0.00	13.80	10.59	0.00
32	29.404	2H-Pyran,tetrahydro-2-[2-propynyloxy]-	140	3.43	13.26	12.39	6.38

The plasma-catalysis reactions in the presence of MCM-41 and ZSM-5 catalysts had the highest deoxygenation rate with 34.6% and 36.6% which was 8.9% and 10.9% higher than plasma reaction at 70 W. The reactions in the presence of Al₂O₃ and TiO₂ contained the lowest deoxygenation degree. As mentioned earlier, MCM-41 and ZSM-5 with high surface area, high porosity, and low dielectric constant provide the appropriate environment for discharge and microdischarge to intensify tar cracking and deoxygenation. Plasma modification can lead to an increase in the number of acid sites on the ZSM-5 catalyst. This effect is likely due to the breakdown of siloxane bonds and the generation of new hydroxyl groups (Si–OH and Al–OH) on the zeolite edges, driven by the reactive species produced by the plasma [51].

Liu et al. [81] investigated catalytic upgrading of anisole in an atmospheric dielectric barrier discharge plasma reactor over Ni-Mo/SiO₂ catalyst, utilizing H₂ as the carrier gas. The products included benzene, toluene, m-xylene, p-xylene, o-xylene, trimethylbenzene, ethyltoluene, phenol, 4-methylanisole, o-cresol, p-cresol, 2,6-dimethylphenol, tetramethylbenzene, and 2,4-dimethylphenol. They found that the deoxygenation rate in the absence of the catalyst in plasma reactor was only 4.6% while in the presence of Ni-Mo/SiO₂ catalyst in the DBD reactor raised this rate to 98% which was attributed the synergistic effect between H₂ plasma and Ni-Mo/SiO₂ catalyst to cleave the refractory C_OR (R = H, or CH_3_) bonds. The DBD plasma initiated the dissociation of H₂ into active high-energy hydrogen species. Blanquet et al. [54], reported formation of alkylated phenols or aromatic compounds, including benzene, phenol, o-cresol, p/m-cresol, guiaicol, 2,4-dimethylphenol, 4-ethylphenol, 4-isopropylphenol and 2- methoxy-4-propylphenol from pyrolysis/non-thermal plasma/catalysis of biomass with a Ni/Al₂O₃ catalyst. Phenol is one of the three main compounds in pyrolysis/non-thermal-plasma-catalysis of RDF at 70 W in the presence of all catalysts. Mequinol produced from MCM-41 and TiO₂ and phenol, 3-methyl produced from ZSM-5 are two phenolic compounds among the three major compounds from plasma-catalysis processing at 70 W.

Fan et al. [21] explored the generation of aromatic hydrocarbons from biomass using DBD-assisted one-stage pyrolysis-catalysis with a zeolite catalyst. They observed that the Ti-modified version of the catalyst demonstrated significant cracking performance, leading to a noticeable increase in light aliphatic hydrocarbons, while the rise in monocyclic aromatic hydrocarbons was attributed to enhanced cracking, which generated more hydrocarbon fragments. Additionally, there was an interaction between the catalyst and the dielectric barrier discharge. The presence of the catalyst was known to influence plasma discharge by strengthening the electric field, altering the discharge type, and promoting micro-discharge formation within the catalyst pores. At the same time, the catalyst exposure to plasma discharge altered its morphology and reduced the metallic oxide catalyst. The discharge also promoted changes in the valence state of Ti ions, which facilitated the production of active radicals, thereby enhancing catalyst activity. Both of these effects contributed to the aromatization process, while the combination of strong acidity and discharge induction helped suppress the formation of polycyclic aromatic hydrocarbons. They suggested that during the DBD-assisted pyrolysis-catalysis process, the plasma would effectively input energy to the chemical bonds of the biomass macromolecules in order to crack and activate the reactant molecules, causing them to radicalise and fragment. Subsequently, these fragments interact with the active site and shape conversion of the zeolite catalyst to induce and arrange the aromatization. Song et al. [15] developed DBD plasma synergistic catalytic pyrolysis for upgrading polyethylene pyrolysis volatiles to yield aromatic-enriched product oil. The aromatization reaction was favoured by both free radicals, which were generated by the plasma, and the specific surface area, acidity, and strength of the catalyst (HZSM-5), which were modified by the plasma. Different sorts of interactions can be induced and influence each other when the catalyst is placed into the plasma discharge zone. The effect of the non-thermal plasma on the HZSM-5 includes modifications to the catalyst physicochemical properties and can also increase the surface chemistry of the HZSM-5. In addition, the presence of the HZSM-5 can impact the plasma, involving changes in electric field distribution and excited species distribution. In later work, Song et al. [82] pyrolyzed waste polyethylene in a dielectric barrier discharge plasma catalytic pyrolysis reactor to produce oil enriched with benzene, toluene, ethylbenzene, and xylene (BTEX). The highest BTEX selectivity (77.04%) and a relatively low coke yield (1.37%) were obtained when the polyethylene to Ga/HZSM-5 catalyst ratio was 2:1, with a discharge power of 20 W. They found that the introduction of non-thermal plasma significantly boosted BTEX selectivity, indicating a synergistic interaction between non-thermal plasma and Ga/HZSM-5 catalyst. When the catalyst is positioned in the NTP discharge zone, micro-discharges form on its surface and within its pores, producing more active species. At the same time, the catalyst characteristics improved, such as a more uniform distribution of Ga species, increased acid site density, and stronger acidity. It was also observed that increasing the discharge power led to a rise in monocyclic aromatic hydrocarbons content, accompanied by a decrease in bicyclic aromatic hydrocarbons content. This was likely due to the higher plasma power producing more short-chain hydrocarbons during the scission reaction of polyethylene or inhibiting the condensation reaction of monocyclic aromatic hydrocarbons.

**Fig. 9:**
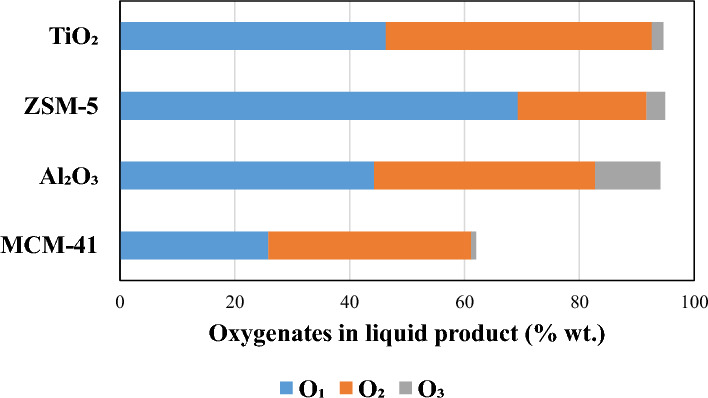
Oxygen atom distribution (% wt.) in the oil products from pyrolysis/non-thermal plasma-catalysis processing at 70 W with MCM-41, Al₂O₃, ZSM-5, and TiO₂

The oxygen atom distribution in the oil products from the pyrolysis/non-thermal plasma-catalysis of RDF with MCM-41, Al₂O₃, ZSM-5, and TiO₂ catalysts at the input power of 70 W is shown in Fig. 9. The oil product in the presence of MCM-41 contained a total of 62% oxygenated compounds with only 0.9% triple-oxygen atom compounds. The oil produced from ZSM-5 had the highest mono-oxygenated proportion with 69.2% and a small proportion of triple-oxygenates with 3.2%. The oxygen distribution in the oil from plasma-catalysis of Al₂O₃ and TiO₂ were almost similar, which could be attributed to their similar BET surface areas. The percentage of triple-oxygenated compounds in the oil from the plasma-catalysis process were lower than the corresponding percentage at the same input power in the absence of catalyst except for Al₂O₃. Fan et al. [83] compared the oxygen atom distribution in bio-oil produced from biomass pyrolysis volatiles through single HZSM-5 catalysis and pre-plasma enhanced HZSM-5 catalysis and reported similar results to those presented here. They reported bio-oil from single step catalysis contained compounds with 57.81% one, 5.49% two, and 1.94% three and above oxygen atoms. Introduction of pre-plasma processing eliminated compounds containing three and above oxygen atoms and decreased mono-oxygenated compounds to 50.36%. Fan et al. [61] investigated the oxygen atom distribution in bio-oil from biomass pyrolysis under catalysis (Zn/HZSM-5) and non-thermal plasma synergistic catalysis (Zn/HZSM-5). They found that the utilization of non-thermal plasma technology notably improved the activation and cracking capabilities of the upgrading process. No organic compounds containing three or more oxygen atoms were detected in the upgraded bio-oils with plasma-catalysis. The content of oxygenated compounds in catalysis exceeded that of the plasma-catalysis, primarily due to limited decomposition efficiency. Partial decomposition of triple-oxygenated compounds led to the formation of some double-oxygenated compounds.

**Fig. 10:**
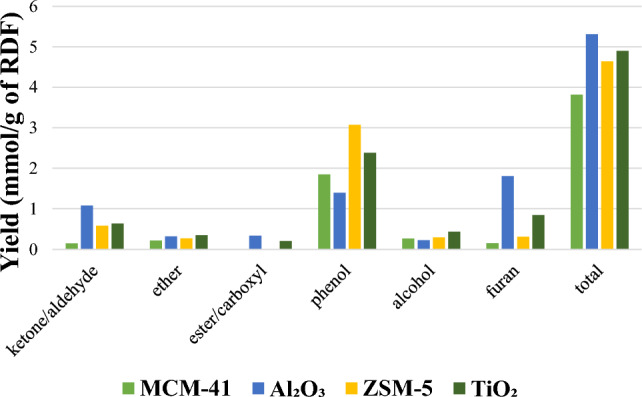
Yield of oxygenated groups (mmol/g of feedstock) in oil products of pyrolysis/non-thermal plasma-catalysis processing of RDF 70 W with MCM-41, Al₂O₃, ZSM-5, and TiO₂

Figure 10 classifies the oil product composition into different oxygenated groups produced from pyrolysis–DBD plasma– catalysis of RDF at 70 W using MCM-41, Al₂O₃, ZSM-5, and TiO₂ as catalysts. In general, there is a direct relation between the high surface area and the yield of low oxygenated oil production for the catalysts used in these experiments. The order of the catalysts in terms of total oxygenated oil yield is MCM-41 < ZSM-5 < TiO₂ < Al₂O₃. The lowest oxygenated oil yield with 3.8 mmol /g_RDF_ was obtained when MCM-41 was applied as the catalyst. This can be linked to the high surface area of MCM-41 (734 m^2^ g^−1^) that resulted in the highest hydrogen production which participated in hydrodeoxygenation reactions. Zeolites provide a larger BET surface area. The conversion of heavy hydrocarbons into light hydrocarbons is affected by cracking, which becomes more efficient when the catalyst material has a higher surface area [84]. Another important factor which effects the efficiency of the catalyst is the pore diameter [84]. MCM-41 has the smallest pore diameter between these four catalysts according to [85]. The dielectric constant impacts catalytic pores and enhances the local electric field when plasma is present. The reaction rate is significantly affected by the electric field, which can be optimized through polarization on the surface of the catalyst pores. Polarization generates an internal electric field that decreases electron energy by opposing the overall electric field. Therefore, while a high dielectric constant reduces the required voltage, it does not improve catalytic reactions in a plasma environment [85]. TiO_2_ and Al_2_O_3_ with higher dielectric constant compared to MCM-41 and ZSM-5 provide more oxygenated groups with higher instability. The highest oxygenated compounds oil yield is related to Al₂O₃ (5.3 mmol /g_RDF_) with the smallest surface area (158 m^2^ g^−1^). This is probably due to the interaction between plasma and catalyst. In non-thermal plasma assisted catalysis reactions, the catalyst dictates the direction of the reactions. Meanwhile, the electric field effectively activates the reactants, transforming them into a plasma state. This process generates free radicals and excited particles, which lowers the activation energy of the reactions. As a result, the rate of adsorption and desorption of reactant molecules on and off the catalyst is improved, thereby enhancing the catalyst’s activity [19]. The presence of a catalyst is recognized to affect plasma discharge through several mechanisms. Firstly, it enhances the electric field, modifies the discharge type, and promotes micro-discharge formation within the catalyst pores. Concurrently, exposure of the catalyst to plasma discharge alters its morphology and decreases the metallic oxide catalyst [68]. Phenols are the main oxygenated group produced from pyrolysis/plasma– catalysis process with MCM-41, ZSM-5, and TiO₂. Phenol production shows the highest yield in the presence of ZSM-5. Ketone/aldehyde production in the presence of catalysts is related to the surface area of catalysts. Ester/carboxyl group were not produced with the plasma-catalysis process with MCM-41 and ZSM-5. Acids, aldehydes, and ketones are known as a source of instability in bio-oil and notable reactivity due to the presence of the carbonyl group (C = O) [61]. In general, it can be concluded that during non-thermal plasma-catalysis processing of RDF, the catalysts with higher surface area such as MCM-41 and ZSM-5 are more beneficial for production of higher value products like phenols and preventing the production of unwanted products such as ketones/aldehydes and ester/carboxyl groups.

Li et al. [86] modified a HZSM-5 catalyst using non-thermal plasma at different voltages for catalytic pyrolysis of biomass. The yield of acids, phenols, and other oxygenates in bio-oil samples exhibited a notable decrease in the presence of the plasma modified catalyst compared to fresh HZSM-5. They suggested that the plasma activated more framework aluminium and increased the mesoporous volume within the HZSM-5. Consequently, a greater number of oxygen-containing components in the pyrolysis gas could be converted into hydrocarbons through the internal pores of the catalyst. Zhao et al. [19], investigated bio-oil upgrading from biomass using vacuum pyrolytic vapours in a non-thermal plasma assisted HZSM-5 catalytic reactor. They performed catalytic and non-thermal plasma/catalytic upgrading and found that the addition of the plasma to the HZSM-5 catalyst led to an increase in aromatics content in the refined bio-oil, indicating promotion of aromatization reactions, also, phenols and alcohols decreased while hydrocarbons increased in yield. Fan et al. [83], investigated bio-fuel production from biomass volatiles by pre-plasma enhanced catalysis with HZSM-5 and Ti/HZSM-5. The implementation of pre-plasma processing led to an enhancement in the total hydrocarbon content, attributed to the plasma induced formation of reactive electrons, ions, free radicals, and excited species. Furthermore, some of the plasma penetrated the catalyst layer along with the reactants, altering the chemical adsorption behavior of the reactants/products on the catalyst surface. This modification enhanced both catalyst activity and reaction selectivity. Fan et al. [87] carried out pyrolysis and non-thermal plasma synergistic upgrading system for lignin with HZSM-5 as the catalyst. The content of monocyclic aromatic hydrocarbons in oil reached 84.34%. They found that the gas–solid discharge on the zeolite facilitated the activation and dissociation of phenols, breaking them into organic fragments that could enter the channels. These fragments then underwent further bond cleavage due to the microdischarges in the pores, leading to the formation of monocyclic aromatic hydrocarbons at the internal acid sites. These monocyclic aromatic hydrocarbons then rapidly escaped due to the plasma sheath’s action.

## Pyrolysis/Non-thermal Plasma/Catalysis of RDF: Mechanism

### Non-thermal Plasma Mechanism

In the presence of only non-thermal plasma, the plasma creates a reaction environment filled with high-energy electrons, free radicals, and excited species, which interact with RDF pyrolysis volatiles. This interaction enhances the breakdown of intermediates derived from RDF and reduces oxygen-containing compounds further. However, the process involves complex chemistry, especially concerning bio-oil upgrading [57]. In a dielectric barrier discharge (DBD) non-thermal plasma, the average electron energy typically ranges from 1 to 10 eV. According to the Maxwellian distribution, an increase in average electron energy results in the generation of more high-energy electrons [88]. The energy in a DBD plasma is sufficient to disrupt most chemical bonds, leading to the formation of ions, free radicals, excited molecules, and energetic electrons [56]. The sequence in which chemical bonds break depends on their binding energy. Breaking down RDF volatiles in a plasma system is complex, but mechanisms for simpler feedstocks, like tar model compounds, have been proposed. Liu et al. [56] suggested a mechanism for toluene reforming in a DBD non-thermal plasma reactor with 75W input power, where toluene decomposes either through direct electron impact or reactions with active species such as •OH, O•, O_3_, and N•. The order of bond breakage is determined by binding energy, with C–H and C–C bonds breaking at different energies. In the plasma reaction, small activated radicals result from breaking C–H, C–O, and O–H bonds. These radicals can recombine to form new compounds, and methyl radicals may decompose further into CH₂, CH, and H radicals within the reactor. Nguyen et al. [53] explored the decomposition of high-density polyethylene (HDPE) in non-thermal plasma and found that its decomposition resembles thermal-only processes. During HDPE decomposition, long-chain volatiles generate carbocations upon collision with energetic species like electrons and N_2_^*^. This reduces the energy barrier for breaking C–H and C–C bonds, leading to more cracking of heavier hydrocarbons into gaseous products.

## Non-thermal Plasma-Catalysis Mechanism

The RDF pyrolysis products which form during the initial pyrolysis stage and are introduced into the plasma-catalytic system are highly complex, making it challenging to establish a clear degradation mechanism. Even with simple single tar model compounds, a variety of hydrocarbon and oxygenated hydrocarbon products can be produced [54]. Adding a catalyst to the discharge area of a DBD plasma system can significantly improve its efficiency. The catalyst shape and surface properties lead to charge accumulation and polarization, which boost the electric field within the plasma. This, in turn, enhances the interaction between the plasma and the catalyst, increasing the chances of reactant collisions and improving surface modifications. The catalyst’s roughness, particularly when it has a high surface area and dense metal sites, further enhances these effects. The synergy between the catalyst and DBD plasma results in reduced carbon deposition, improved conversion, selectivity, stability, better catalyst reusability, and increased energy efficiency [55]. The synergy between plasma and catalyst in a reaction can be understood in two main ways. First, the plasma and the catalyst influence the gas conversion independently: the plasma induces chemical reactions while the catalyst lowers the activation energy required. Second, their interaction together enhances the process. The rough and porous surface of the catalyst can amplify the electric field, impacting electron density and increasing electron impact dissociation and ionization rates. This changes the discharge from filamentary to a mix of surface discharges, boosting reaction rates. Reactants can also adhere to the catalyst, extending their time in the reactor and improving conversion rates. Additionally, the plasma can alter the catalyst’s properties by increasing adsorption and surface area. Strong microdischarges can create hot spots, locally heating the catalyst. Despite the overall reactor temperature being relatively low (e.g., 250 °C), these local hot spots can achieve higher temperatures, facilitating efficient thermal catalytic reactions [60]. Fan et al. [83] proposed a mechanism for biomass pyrolysis volatiles upgraded through a series of reactions influenced by the interaction between plasma discharge and catalyst conversion. The plasma, generating both short-lived and long-lived species, plays a significant role in enhancing catalysis. The high-voltage electric field activates the pyrolysis volatiles, resulting in the formation of highly reactive species. These reactive species provide the foundation for catalytic reactions, while the long-lived plasma affects the chemical adsorption behaviour on the catalyst, improving catalyst activity and reaction selectivity. During the process, intermediate oxygenates diffuse into the catalyst’s pores, where they undergo further breakdown and reforming through various reactions such as cracking, decarbonylation, decarboxylation, dihydroxylation, oligomerization and aromatization, leading to the formation of smaller hydrocarbon fragments that contribute to a hydrocarbon pool. Titanium ions, due to their strong polarization ability, promote cracking, deoxygenation, and aromatization. The valence change of titanium ions and electron migration also enhance carbonium ion reactions. The overall conversion process involves catalyst activation, catalytic reactions, and related interactions, ultimately producing aromatics through protonation, cyclization, dehydrogenation, and aromatization. Some of these aromatic hydrocarbons may further react to form polycyclic aromatic hydrocarbons. However, due to incomplete reactions, some oxygenate compounds, particularly hydroxyl-containing ones, remain, as their high hydrophilicity makes them difficult to fully eliminate. Xiao et al. [51] compared a synergy mechanism of plasma-catalysis pyrolysis with conventional catalytic pyrolysis. Large quantities of heavy hydrocarbon intermediates struggle to penetrate the internal micropores of the catalyst during pyrolysis with the catalyst alone, leading to pore blockage from coke deposition. This results in limited aromatization due to the inaccessibility of acid sites on the catalyst. In contrast, plasma-enhanced pre-cracking breaks down heavy intermediates into lighter ones that can diffuse into the internal pores of ZSM-5. The combined effect of plasma and catalysis during pyrolysis includes plasma’s impact on volatiles and its modification of the catalyst. Plasma generates highly energetic electrons with energy levels exceeding the bond dissociation energies of polypropylene (C–H = 415 kJ/mol and C–C = 331 kJ/mol), thus facilitating pre-cracking. Additionally, plasma alters the acid sites and defects on the catalyst surface, improving its performance.

## Pyrolysis/Non-thermal Plasma/Catalysis of RDF: Used Catalyst Characterisation

**Fig. 11:**
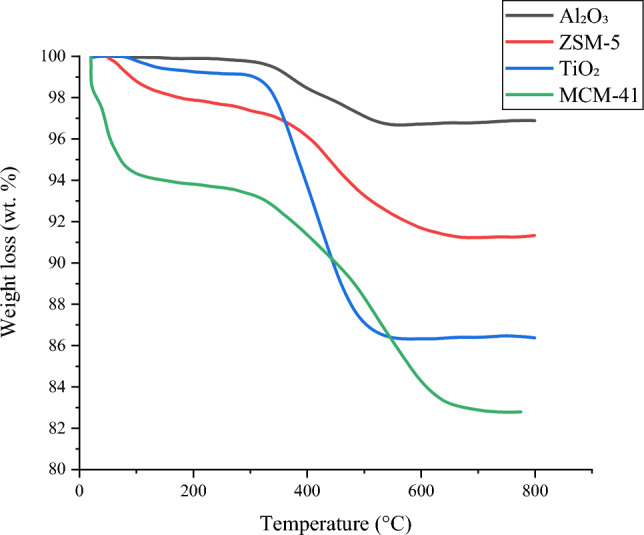
TPO thermograms of the used Al₂O₃, ZSM-5, TiO₂_,_ and MCM-41 catalyst materials

Temperature programmed oxidation (TPO) was employed to quantify the amount of carbonaceous coke accumulated on the catalysts used in the pyrolysis/non-thermal plasma/catalysis process at 70 W plasma input power. Figure 11 shows the TPO thermograms of the used Al₂O₃, ZSM-5*,* TiO₂_*,*_ and MCM-41 catalysts. The highest amount of carbon deposition was observed with the TiO₂ catalyst at 13.1 wt%. The percentage of carbon deposited on MCM-41, ZSM-5, and Al₂O₃ catalysts were 11.9 wt%, 6.7 wt%, and 3.0 wt%, respectively. For zeolite catalysts, due to the strong acidity, coke deposition on the surface is unavoidable during the cracking of volatiles [53]. The oxidation of the carbon in the catalysts were complete at the TPO temperatures lower than 550 °C for all the used catalysts.

Blanquet et al. [23] carried out TPO for the spent catalysts from the pyrolysis-plasma/catalysis process of biomass using various metal-promoted catalysts. They linked the observed weight loss to the oxidation of carbon, indicating carbon deposits ranging from 5 wt% to 7 wt% on the used catalysts. Zeng et al. [89] studied plasma/catalysis of biogas with different metal promoters (K, Ce, and Mg) on a Ni–Al₂O₃ catalyst, reporting comparable carbon deposition levels of 6.54 wt%, 6.84 wt%, and 6.59 wt% for Mg-Ni–Al₂O₃, Ce-Ni–Al₂O₃, and K-Ni–Al₂O₃ catalysts, respectively. Xiao et al. [51] investigated coke formation on ZSM-5 and figured out plasma-catalysis resulted in less coke deposition compared to the non-plasma catalytic process. Additionally, the plasma presence increased the number of acid sites on the catalyst and facilitated C–H bond cleavage, which can enhance cyclization and polymerization through the dehydrogenation of hydroaromatic compounds and the expansion of the skeletal carbon network.

It has been reported [54] that compared to conventional catalysis, using non-thermal/plasma catalysis produces lower deposition of carbonaceous coke on the catalyst. During plasma catalysis, the environment surrounding the catalyst reaches a higher temperature compared to when the catalyst operates alone. This temperature increase is attributed to excited molecules colliding with energetic electrons, resulting in energy transfer between the electrons and the heavier molecules. The higher temperature induced by plasma enhances carbon reactions, thereby reducing catalyst coke formation [54]. Meng et al. [90] claimed that the catalysts in non-thermal plasma-catalysis can help stabilize radicals and prevent them from undergoing excessive C–H bond dissociation, which could otherwise lead to the formation of coke. Blanquet et al. [23] hypothesized that the Boudouard reaction’s role in plasma-catalysis results in reduced coke deposition compared to catalysis alone. TPO analysis of catalyst carbon deposits can differentiate between various types of carbon deposits: amorphous carbons, which undergo oxidation at lower temperatures (< 550 °C), and more graphitic filamentous carbons, which oxidize at higher temperatures (> 550 °C) [91]. TPO thermograms showed that the carbon deposited on the surface of all the used catalysts was mainly amorphous, there was also graphitic type carbon on the surface of MCM-41 and Al₂O₃.

Carbon deposits on catalysts can significantly affect their performance in various reactions. Both amorphous and graphitic carbon can form, each impacting catalyst deactivation differently. Amorphous carbon is typically less harmful, as it can be readily hydrogenated under reaction conditions [92]. In contrast, graphitic carbon is more stable and can coat catalyst surfaces, leading to deactivation [93]. The formation of carbon deposits involves a formation-diffusion/elimination process, influenced by factors such as catalyst particle size and metal-support interactions [92]. In some cases, graphitic carbon can selectively block certain reaction sites, altering product distributions and selectivities [92]. The impact of carbon deposits varies with the type of reaction and catalyst composition. For example, in oxidation reactions, nitrogen-functionalized carbons can enhance catalyst deactivation [94]. In hydrocarbon reactions, both graphitic and amorphous carbon can lead to nonselective deactivation and suppress specific catalytic activities [95]. Wang et al. [93] studied the impact of different carbon residues on the deactivation of Ni–CaO–ZrO_2_ catalysts in CH_4_ dry reforming. The claimed that catalyst deactivation may not directly correspond to the quantity of carbon formed, as the individual characteristics of the carbon residues might have a more significant impact. The formation of amorphous carbon species does not always result in catalyst deactivation. Instead, deactivation was observed to be strongly associated with the formation of graphite carbon, which can coat the catalyst surface. They observed that the amorphous carbon has a highly disordered structure and thus may have a minimal negative effect on the active sites. It is particularly notable as an active intermediate species during the carbon dioxide reforming of methane reaction and can also act as a precursor for various other types of carbon deposition, such as whisker, encapsulating, and graphite carbon [93].

**Fig. 12:**
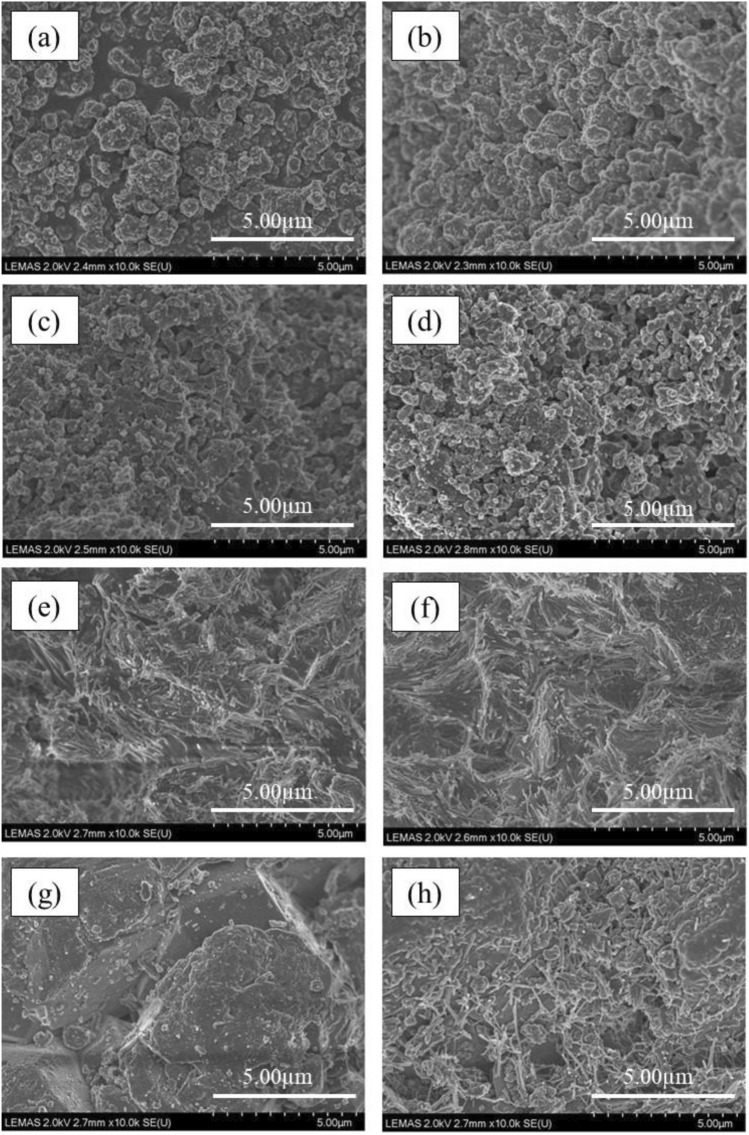
SEM images showing the surface morphology of the fresh and used catalyst materials **a** fresh TiO₂, **b** used TiO₂, **c** fresh MCM-41, **d** used MCM-41, **e** fresh ZSM-5, **f** used ZSM-5, **g** fresh Al₂O₃, **h** used Al₂O₃

Figure 12 shows the scanning electron micrographs of the fresh and used catalysts. The SEM images of fresh and used catalysts were compared to evaluate deposition of carbon on the surface of catalysts during the reaction. SEM observations reveal the presence of filamentous carbons generated on the Al₂O₃ catalyst (Fig. 12h). These carbons appear as long, smooth, and thin filaments distinctly visible on the reacted Al₂O₃ catalyst. There is no observable difference on the surface between the unreacted and reacted TiO₂, MCM-41, and ZSM-5 catalysts (Fig. 12b, d, and f).

SEM image of MCM-41 shows the spherical shape of the catalyst structure. When used as catalysts in chemical reactions, spherical structures offer several advantages: they exhibit high mechanical strength, provide short diffusion pathways for substances, enhance dispersion through electrostatic charge stabilization, and have a high surface area relative to their volume [53], which is also confirmed by high BET surface area of MCM-41. Xiao et al. [51] found that the fresh ZSM-5 catalyst had a smooth, flaky surface that became rougher after plasma modification. After using the catalyst alone, the surface was covered with coral-like substances. In contrast, plasma-catalysis resulted in only a few small agglomerated particles on the surface and pores of the spent catalyst. Polypropylene’s low molecular weight and small size make it difficult to enter deep into the zeolite pores without pre-cracking. Plasma-catalysis helps by pre-cracking the polymer, allowing it to better penetrate the catalyst’s pore network and improve aromatization, whereas non-plasma catalysis leads to pore blockage by dense agglomerates.

## Conclusions

A two stage pyrolysis/non-thermal plasma/catalysis reactor system has been used to produce upgraded oil and gas from refuse-derived fuel (RDF). A series of experiments were carried out over a range of input powers without a catalyst. The introduction of non-thermal plasma and increasing the power led to higher gas yield compared to pyrolysis alone. The yield of H₂, CO, CO₂, tended to increase with increased input plasma power. Higher yields of CO and CO₂ showed deoxygenation of oxygenated hydrocarbons occurred during the plasma process through decarboxylation and decarbonylation. The degree of deoxygenation was dependent to the plasma input power rising to 25.7% for the input plasma power of 70 W. The oil produced at highest input powers contained the lowest proportion of oxygenated compounds with three oxygen atoms, containing only 6.7% at 70 W.

A second series of experiments were conducted with different catalysts introduced into the non-thermal plasma reaction zone, using TiO₂, MCM-41, ZSM-5, and Al₂O₃ catalysts. The MCM-41 and ZSM-5 catalysts promoted higher gas production, while TiO₂ and Al₂O₃ resulted in higher oil yield compared to the non-thermal plasma at 70 W in the absence of catalyst. Utilization of MCM-41 and ZSM-5 along with plasma at 70 W generated the highest yield of CO, CO₂, and H₂ due to the synergy between catalyst and plasma. The lower yield of gas compounds produced from plasma-catalysis of Al₂O₃ and TiO₂ could be attributed to the discharge characteristics of the catalysts materials such as smaller surface areas. The highest degree of deoxygenation was 34.6% and 36.6% obtained during pyrolysis/non-thermal plasma/catalysis with MCM-41 and ZSM-5 at 70 W input power. The catalysts ranked in terms of total oxygenated oil yield are as follows: MCM-41 < ZSM-5 < TiO₂ < Al₂O₃. This ranking can be attributed to the surface area of the catalysts which in turns influences the plasma discharge characteristics of the different materials. MCM-41 and ZSM-5 with highest surface areas resulted in a high hydrogen production, contributing to hydrodeoxygenation reactions. MCM-41 and ZSM-5 with the highest surface area promoted phenol production and suppressed formation of ketone/aldehyde and ester/carboxyl groups which are the source of instability in bio-oil. In the presence of MCM-41, the oil product comprised 62% oxygenates, with only 0.9% consisting of compounds with three oxygen atoms. Conversely, the oil produced from ZSM-5 exhibited the highest proportion of mono-oxygenated compounds at 69.2%, along with a small proportion of triple-oxygenated compounds at 3.2%.

## Data Availability

Data will be made available on reasonable request.
